# RNA G-quadruplexes regulate mammalian mirtron biogenesis

**DOI:** 10.1016/j.jbc.2025.108276

**Published:** 2025-02-07

**Authors:** Uzma Salim, Manoj B. Menon, Sonam Dhamija, Perumal Vivekanandan

**Affiliations:** 1 Kusuma School of Biological Sciences, Indian Institute of Technology, New Delhi, India; 2 Integrative and Functional Biology Unit, CSIR - Institute of Genomics and Integrative Biology, New Delhi, India; 3 Academy of Scientific and Innovative Research (AcSIR), CSIR - Institute of Genomics and Integrative Biology, New Delhi, India

**Keywords:** G-quadruplex, RNA G-quadruplex(rG4), mirtrons, splicing, miRNA, noncanonical miRNA, Drosha-independent processing

## Abstract

Mirtrons are a predominant class of noncanonical microRNAs derived from introns through a Drosha-independent, splicing-dependent pathway. Unregulated splicing of introns containing hairpins may adversely impact Dicer/Ago-mediated canonical microRNA biogenesis. However, the mechanism regulating mirtron biogenesis remains poorly understood. We found that the 5′ arm of plant mirtrons and invertebrate mirtrons are enriched for uracils; in contrast, the 5′ arm of vertebrate mirtrons are enriched for guanines. Further analysis revealed that most of the mammalian mirtrons contain an RNA G-quadruplex (rG4); this was not observed among plant/invertebrate mirtrons. Interestingly, almost all the rG4s in mammalian mirtrons were present in the 5′ arm. Predicted rG4s in human mirtrons form a G-quadruplex structure *in vitro* and rG4 formation in the 5′ arm of mirtrons facilitates splicing-mediated biogenesis of mirtrons. Notably, the disruption of rG4s in the 5′ arm of mirtrons inhibits splicing and maturation; while mutations outside the rG4-motif do not impact mirtron biogenesis. Our findings support the notion that rG4s at the 5′ arm are key regulatory elements in the evolutionary landscape of mammalian mirtrons. This work advances our current understanding of mirtron biogenesis and highlights additional roles for rG4s in small RNA biology.

MicroRNAs (miRNAs) are a major class of small noncoding RNAs that have emerged as key regulators of gene expression with significant implications in various biological processes and disease pathologies (1, 2). They have also been implicated as promising diagnostic markers and therapeutic targets in human health and disease (3, 4). The biogenesis of miRNAs is a multitiered procedure initiated with transcription by RNA polymerase II or III, forming primary miRNA (5, 6). The primary miRNA is acted upon by the microprocessor complex, comprising Drosha and cofactor DGCR8, to generate precursor miRNA (pre-miRNA) (7, 8, 9). Subsequently, pre-miRNAs are transported from the nucleus to the cytoplasm by exportin-5 (10, 11). Dicer acts on pre-miRNA in the cytoplasm, generating a mature miRNA duplex (12). The thermodynamically stable strand of the duplex is then loaded onto the RNA-induced silencing complex, guiding RNA-induced silencing complex to target mRNAs through partial complementarity with the miRNA seed region in the 3′ UTR of the target mRNA (13, 14, 15). This binding can lead to translational repression and/or mRNA degradation, ultimately suppressing gene expression (16, 17).

A fraction of these miRNAs is synthesized through diverse noncanonical pathways. Mirtrons, a predominant class of noncanonical miRNAs, originate from the introns of the protein-coding genes. Mirtrons arise from a unique Drosha-independent, splicing-dependent pathway to form precursor hairpin structures (pre-miRNA) (18, 19). Their biogenesis starts with the transcription of protein-coding genes that contain introns. The spliceosome binds to the 5′ and 3′ splice sites located at the corresponding ends of the short hairpin intron and splicing results in the formation of a lariat. Subsequently, the lariat debranching enzyme (DBR1) facilitates the conversion of the lariat into a short hairpin structure, known as the mirtron, which mimics the precursor hairpin of canonical miRNAs. Mirtrons are exported to the cytoplasm and further processed by Dicer to produce mature miRNA targeting specific mRNAs for posttranscriptional regulation (20).

Depending on the location of splice sites relative to the hairpin termini, mirtrons are categorized into three classes: conventional mirtrons, 5′-tailed mirtrons, and 3′-tailed mirtrons. While conventional mirtrons have the 5′ and 3′ splice sites placed precisely at the termini of the hairpin, the 5′ and the 3′-tailed mirtrons have a “tail” of extra nucleotides at their respective termini. These mirtrons require another step of degrading the tail to form a pre-miRNA. The 3′-tailed and 5′-tails are excised by an RNA exosome and an unidentified exonuclease, respectively (21).

Comprising nearly 15% of the total miRNA population, mirtrons have been shown to play critical roles in various biological processes and have implications for human health and disease. They contribute to fine-tuning gene expression during development, cell proliferation, cell differentiation, angiogenesis, and neurogenesis (21, 22, 23, 24, 25, 26, 27). Dysregulation of mirtrons has also been linked to various diseases, including cancer and neurological disorders, thereby providing an additional layer of complexity in gene regulatory networks (23, 28, 29, 30, 31). Continued research in this field is likely to reveal mirtron functions and regulatory mechanisms and their potential as therapeutic targets or diagnostic markers in various diseases (19, 23, 31, 32, 33).

G-quadruplexes (GQs) are noncanonical nucleic acid secondary structures formed by guanine-rich sequences, typically found in telomeres and other genomic regions (34, 35, 36). GQs play a pivotal role in various cellular processes, including telomere maintenance, DNA replication, transcription, translation, splicing, genome stability, gene regulation, and epigenetic modifications, with several GQ-binding ligands being investigated as potential anticancer leads (37, 38, 39, 40, 41, 42, 43, 44, 45, 46, 47, 48, 49, 50, 51, 52, 53, 54, 55, 56, 57, 58, 59, 60, 61). GQs consist of stacked guanine tetrads, where four guanine bases form a planar arrangement stabilized by Hoogsteen hydrogen bonding (62). A putative GQ-forming sequence consists of four or more G-tracts separated by loop sequences. The G-tracts consist of two or more guanine residues, following the pattern 5′-GGN_1-7_GGN_1-7_GGN_1-7_GG-3′ (representing a two tetrad GQ with a maximum loop length of seven nucleotides) (36). Monovalent cations like K^+^ and Na^+^ aid in the stabilization of GQ structures by neutralizing the electrostatic repulsion arising from the negative charge of guanine's C6 oxygen (63). RNA G-quadruplexes (rG4s) are formed more readily than DNA GQs due to the single-stranded nature of RNA (64). Generally, rG4s exhibit enhanced thermodynamic stability attributed to the presence of the 2′-hydroxyl group. Reports suggest that rG4s can influence splice site selection, exon skipping, and alternative splicing events, potentially modulating gene expression (49, 51, 52, 65, 66, 67). They can form within intronic regions, particularly near splice sites, and their presence can either enhance or hinder splicing efficiency by affecting the recruitment of spliceosome components. Their regulatory effects appear context-dependent, varying based on the specific sequence, location, and surrounding RNA and protein factors.

GQs are enriched at splice sites, and several RNA-binding proteins bind to GQs and modulate alternative splicing (66). In addition, GQs are known to impact the Drosha-mediated processing of a few miRNAs (68). Here, we identify the enrichment of rG4s in the 5′ arm of mirtrons and demonstrate a critical role for rG4s in regulating splicing-dependent biogenesis of mirtrons. Our findings highlight how rG4 formation may be a previously unrecognized additional layer of control to regulate splicing-derived pre-miRNA mimics that are processed by the cytoplasmic miRNA processing machinery.

## Results

### Human mirtrons are enriched for 5′ rG4

**Figure 1:**
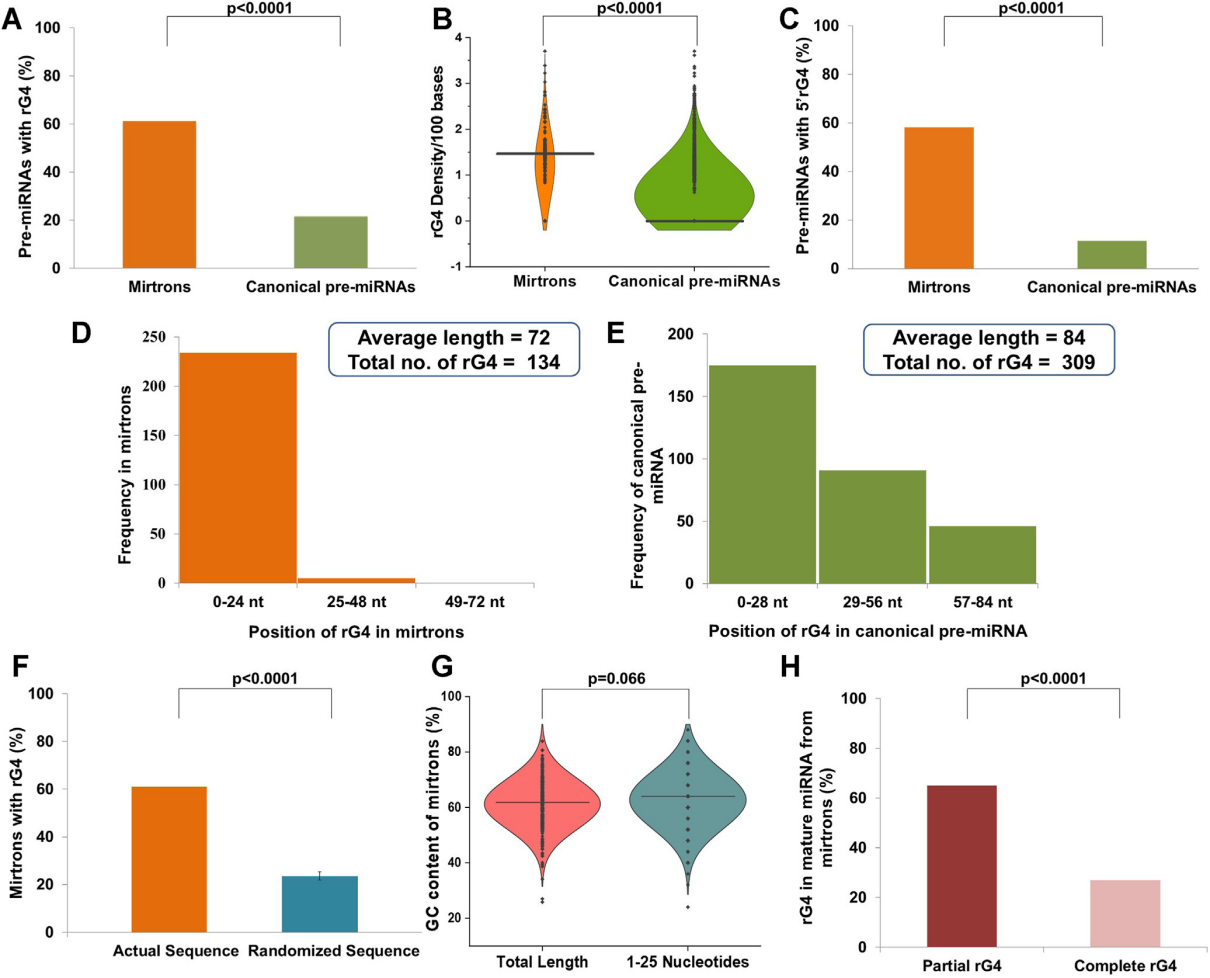
**Preferential enrichment of rG4s in human mirtrons.***A*, bar graph showing the presence of rG4s in mirtrons (n = 239) and canonical pre-miRNAs (n = 1453). About 61% of mirtrons possess at least one rG4 while only about 22% of canonical pre-miRNAs have rG4. *B*, violin plots showing the distribution of rG4 density/100 bases in mirtrons (n = 239) and canonical pre-miRNAs (n = 1453). Mirtrons have significantly higher rG4 density compared to canonical pre-miRNAs. *C*, bar graph showing the presence of rG4s in the 5′ arm of mirtrons (n = 239) and canonical pre-miRNAs (n = 1453). About 58% of mirtrons have the rG4 positioned in the 5′arm, while only about 11.5% of canonical pre-miRNAs have rG4 located in the 5′arm of the pre-miRNA sequence. *D*, histogram representing the frequency of position of rG4s in mirtron sequences calculated with respect to the average total length of the pre-miRNA sequence. *E*, histogram representing the frequency of position of rG4s in canonical pre-miRNA sequences calculated with respect to the average total length of the pre-miRNA sequence. *F*, bar graph showing the presence of rG4s in native and randomized sequences of human mirtrons. *G*, violin plots showing the GC% of mirtrons (n = 239) in the whole pre-miRNA sequence and 1 to 25 nucleotides of the mirtron. *H*, bar graph showing the presence of rG4 in mature miRNA formed from mirtrons. miRNAs, microRNAs; rG4, RNA G-quadruplex.

We downloaded 1453 human canonical pre-miRNA and 239 human mirtron sequences from miRBase (https://mirbase.org/) and analyzed them using the QGRS Mapper online software (https://bioinformatics.ramapo.edu/QGRS/index.php) for potential rG4 forming sequences with loop length ranging from 1 to 7 nucleotides {(G≥2X1-7)}; this will allow us to pick up all nonoverlapping rG4s with two or more tetrads. We compared the rG4 distribution in mirtrons with canonical pre-miRNAs. Our results show that out of 239 mirtrons, ∼61% (n = 145) contain at least one putative rG4 in their sequence (Table S1), while only ∼22% (n = 320) of 1453 canonical pre-miRNAs contain at least one putative rG4 in their sequence (Fig. 1*A*). Human pre-miRNAs have been reported to be enriched with rG4s (68). However, rG4 density (number of rG4 per hundred bases) observed in mirtrons is significantly higher than that in canonical pre-miRNA sequences (Fig. 1*B*; median for mirtrons 1.47 *versus* canonical pre-miRNA 0; *p* < 0.0001). We then mapped the positions of rG4s within mirtrons and canonical pre-miRNAs. The rG4s within canonical pre-miRNA sequences are evenly distributed along the length of the pre-miRNA. In contrast, over 58% of all rG4s in mirtrons are concentrated at the 5′ arm (*i.e.*, 1–25 nucleotides) (Fig. 1, *C*–*E*). The rG4 density is significantly lower in randomized mirtron sequences, suggesting that the enrichment (of rG4s) is not explained by the mononucleotide composition or the GC content (Fig. 1*F*; *p* < 0.0001). The GC content of the 5′ arm of mirtrons is comparable to that of the entire mirtron, suggesting that the enrichment of rG4s in the 5′ arm is not linked to differences if any, in the nucleotide composition (Fig. 1*G*). Interestingly, the majority of rG4s in mirtrons (∼65%) are disrupted during their maturation to form miRNA (Fig. 1*H*), suggesting a potential role for these nucleic acid secondary structures in mirtron processing or splicing.

### Enrichment of rG4s in the 5′ arm is limited to vertebrate mirtrons

**Figure 2:**
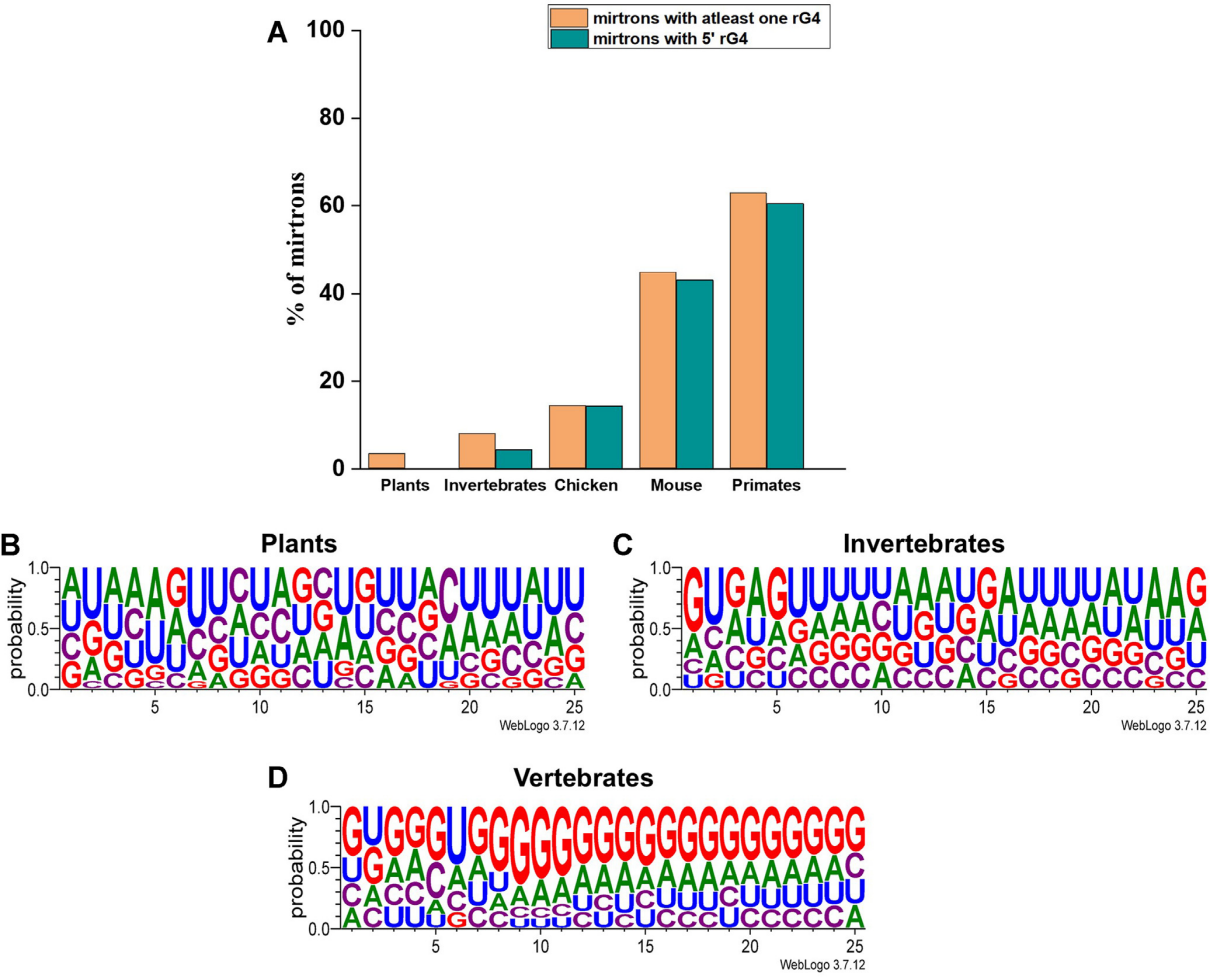
**rG4s in mirtrons across taxonomic groups**. *A*, mirtrons harboring at least one rG4 motif within their sequence are notably low in plant species but show an increasing trend with the rising complexity of organisms within the animal kingdom. Among the vertebrates, majority of primate mirtrons comprising of mirtron sequences from rhesus macaques, chimpanzees, and humans, contain rG4 motifs. Intriguingly, almost all the rG4s within the mirtron sequence in primates are situated in the 5′ arm; this phenomenon was not observed among plant and invertebrate mirtrons. *B* and *C*, nucleotide composition of the 5′ arm (first 25 nucleotides) (*B*) plant mirtrons (n = 30) and (*C*) invertebrate mirtrons (n = 105) shows an enrichment of Uracils (Us), while (*D*) vertebrate mirtrons (chicken, mouse, and primates) (n = 748) show an enrichment of Guanines (Gs). rG4, RNA G-quadruplex.

We analyzed all validated mirtron sequences (n ≥ 5) from different organisms using the QGRS Mapper tool for potential rG4 forming sequences and their position with loop length ranging from 1 to 7 {(G≥2X1-7)}. Our results reveal a positive correlation between the presence of rG4s within mirtrons and the evolutionary hierarchy of organisms. The abundance of rG4-containing mirtrons varies greatly across different taxonomic groups. Only a small fraction (<10%) of mirtrons from plants (*Manihot* *esculenta, Oryza sativa, and Medicago truncatula*) and invertebrates (*Caenorhabditis elegans, Drosophila pseudoobscura, Drosophila simulans, and Drosophila melanogaster*) contain rG4 motifs. A higher proportion of mirtrons from chicken and mouse contain rG4 motifs. Over 60% of mirtrons from primates, including rhesus macaque, chimpanzee, and humans, contain a rG4 motif (Fig. 2*A*). Interestingly, almost all of the rG4s in mirtrons are located within their 5′ arm in vertebrates ranging from chicken to primates. In contrast, none of the rG4 motifs in plant mirtrons were located in the 5′ arm, while this proportion increased marginally in invertebrates (Fig. 2*A*). In other words, our findings suggest that enrichment of rG4 motifs in the 5′ arm of mirtrons is a unique feature of vertebrate mirtrons.

We then analyzed the nucleotide composition of the first 25 nucleotides (5′ end) of mirtrons from plant, invertebrate, and vertebrate genomes. The sequences of the first 25 nucleotides (5′ end) from all mirtrons within each taxonomic group were aligned to evaluate the occurrence and likelihood of each nucleotide. The weblogos (Fig. 2, *B* and *C*) indicate a significant enrichment of uracil (U) at several positions in the first 25 nucleotides (5′ end) among plant mirtrons and invertebrate mirtrons. Interestingly, among vertebrate mirtrons (including chicken, mouse, and primates), 23 out of the first 25 nucleotides (5′ end) are enriched for guanine (G) (Fig. 2*D*).

### Biophysical characterization of the putative rG4 sequences from human mirtrons

**Figure 3:**
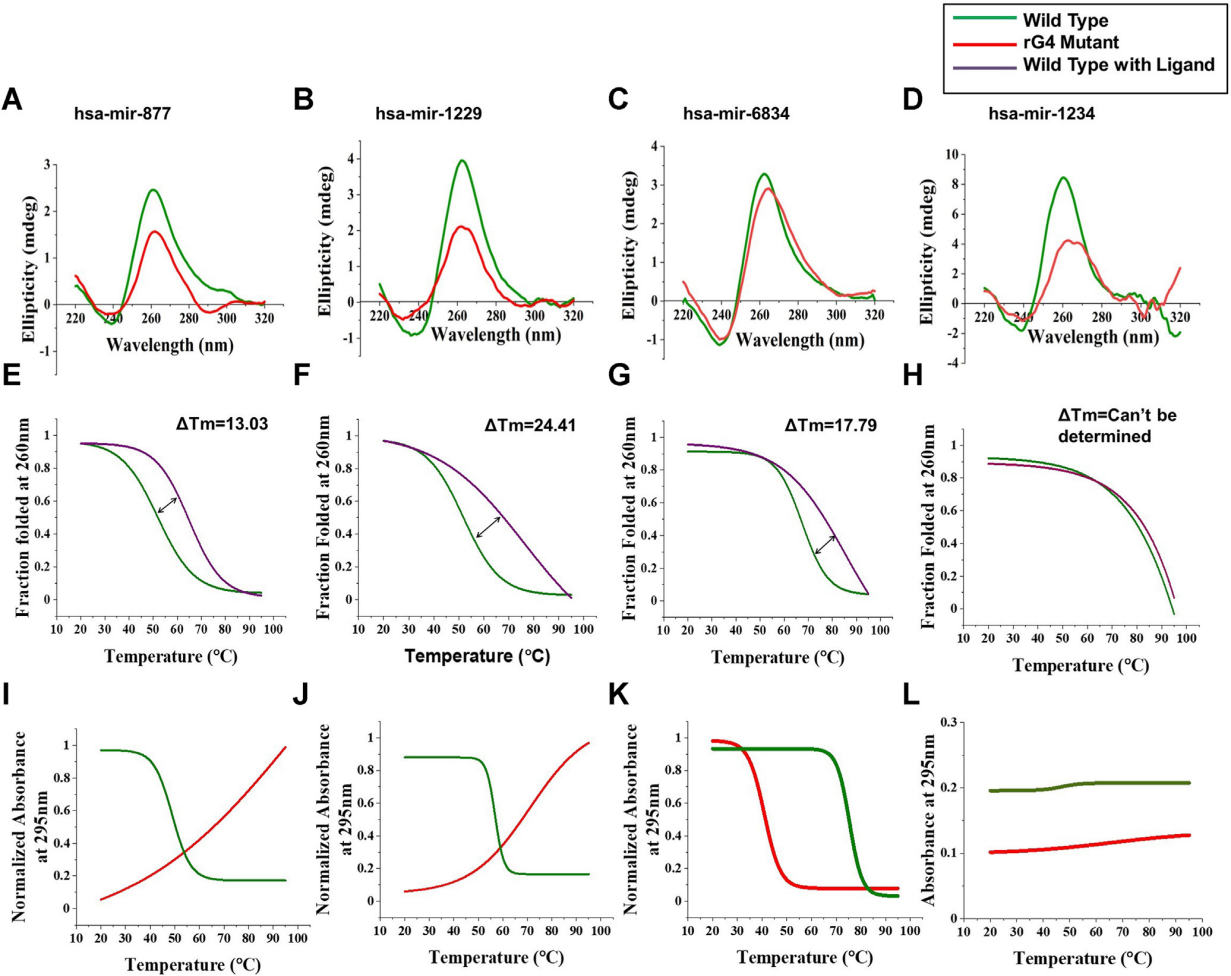
**Biophysical analyses of mirtron-derived rG4s**. *A–D*, CD spectroscopy showing the formation of parallel G-quadruplexes by the RNA oligonucleotides from mirtrons (n = 4) with different types of rG4 motif. The rG4 mutant RNA oligonucleotide does not form GQ structures*. E–H*, CD melt curves showing BRACO19-mediated stabilization of the rG4 in the RNA oligonucleotides from mirtrons. *I–L*, UV melt curves for rG4 mirtron RNA oligonucleotides obtained by monitoring UV absorbance at 295 nm as a function of temperature (20–95 °C). CD, circular dichroism; GQ, G-quadruplex; rG4, RNA G-quadruplex.

To determine the topology of the rG4s, we randomly selected four human mirtrons: one with single rG4 (a 2-tetrad rG4 {hsa-miR-877}, two 3-tetrad rG4s {hsa-miR-1229 and hsa-miR-6834}, and one 4-tetrad rG4 {hsa-miR-1234}) motif so results can be related to 2-, 3-, and 4-tetrad rG4 motifs at the 5′ arm of mirtrons. The sequences of the mirtrons were mutated to disrupt rG4 formation without potentially impacting the stem-loop hairpin structure. To determine if the predicted rG4s form GQs *in vitro*, we performed circular dichroism (CD) spectroscopy with RNA oligonucleotides for the selected putative rG4s at the 5′ arm of human mirtrons and their respective mutants. The RNA oligonucleotide sequences of wildtype and rG4 mutant are listed in Table S2. The CD spectra analysis revealed that all four mirtron-derived rG4s formed parallel GQs *in vitro*, characterized by a prominent positive peak around 260 nm and a negative peak at approximately 240 nm. As expected, the mutations within the rG4 sequences (rG4 mutants) that may form transient secondary structures exhibited lower ellipticity values and a shift in the CD spectra, indicating a decrease in the rG4 population (Fig. 3, *A*–*D*). These results strongly support the GQ formation of the four wildtype rG4 sequences of mirtrons and verify the inability of the rG4 mutants to form such defined secondary structures. Melt curve analysis was also conducted at the wavelength with the highest peak in the spectra (264 nm). The CD spectra of the rG4 mutant of the mirtron hsa-mir-1234 clearly show altered 260 nm positive and 240 nm negative peaks, indicating the disruption of the rG4 motif due to the mutation. In contrast, the CD spectra of the other three mirtrons—hsa-mir-877, hsa-mir-1229, and hsa-mir-6834—exhibit reduced ellipticity but do not display significant alterations in peak characteristics compared to the wildtype rG4 sequence. To further investigate this, we conducted a CD melting analysis on the wildtype and mutant sequences of these mirtrons, confirming that mutations in the rG4 structure led to its disruption. The rG4 mutants exhibited a significantly lower melting temperature (T_m_) compared to the respective wildtype oligonucleotides. Furthermore, upon adding 10 μM BRACO19, a GQ stabilizing ligand, the rG4 mutants showed no significant change in their melting temperature (Fig. S1). In contrast, the wildtype oligonucleotides exhibited a substantial increase in T_m_. The addition of BRACO19 resulted in an increase in the melting temperature and a right shift in the melt curve, indicating increased stability of the rG4 structures (Fig. 3, *E*–*H*). Furthermore, the rG4 wildtype RNA oligonucleotides and their respective rG4 mutants were subjected to UV melting (20–95 °C) at 295 nm. During the melting process, the UV absorbance of wildtype rG4 oligonucleotides exhibited a characteristic decline, indicative of a hypochromic shift. Some rG4 mutant oligonucleotides exhibited a hyperchromic curve, characteristic of single-stranded RNA, while others showed a hypochromic curve with a significantly lower T_m_ compared to the wildtype oligonucleotides. The 4-tetrad rG4 oligonucleotide (hsa-mir-1234) did not melt even at 95 °C (Fig. 3, *I*–*L*). Taken together, our biophysical analyses clearly demonstrate that the putative rG4 sequences from the 5′ arm of mirtrons form parallel rG4 structures *in vitro* which are stabilized by BRACO-19.

### rG4s in the 5′ arm of mirtrons facilitate splicing

**Figure 4:**
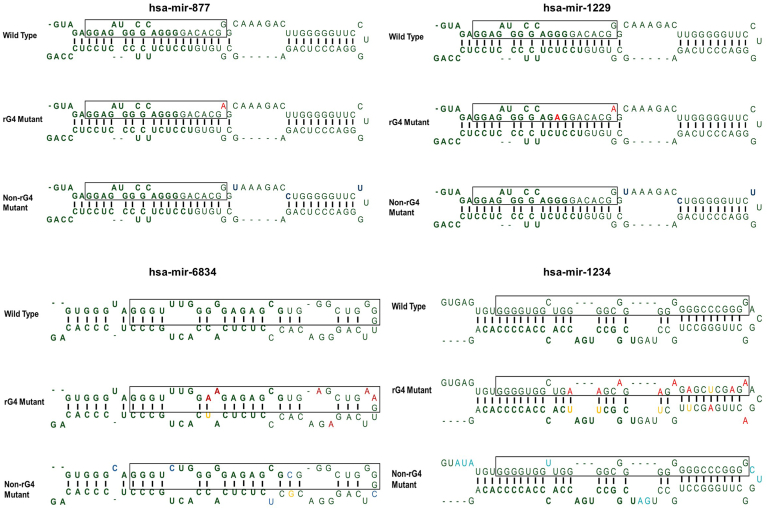
**Diagrammatic representation of stem-loop structures of wildtype, rG4 mutant, and non-rG4 mutant of selected human mirtrons**. rG4 mutations are marked in *red*, non-rG4 mutations are marked in *blue*, and complementary mutations made to maintain the stem-loop structure are marked in *yellow*. rG4, RNA G-quadruplex.

**Figure 5:**
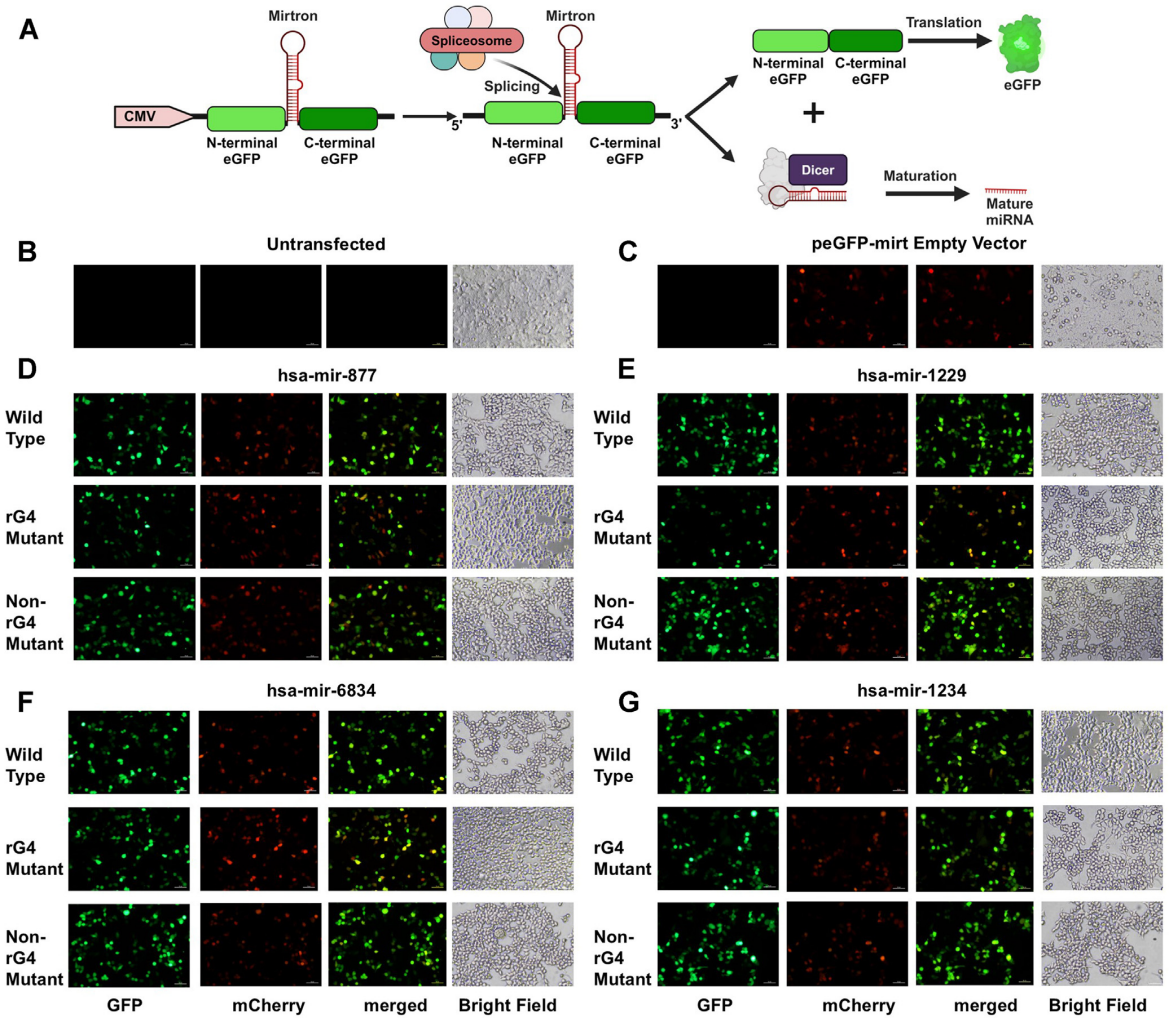
**GFP splicing reporter for mirtron biogenesis**. *A*, schematic representation of pEGFP-Mirt cloning strategy. *B–G*, fluorescence microscopy for GFP expression on appropriate splicing of mirtrons from the intronic region between artificial exons encoding the N- and C-terminal regions of eGFP in pEGFP-Mirt vector transfected in HCT116 wildtype cell line. Red fluorescence from cotransfected pcDNA-mCherry is shown as an internal control. *D*–*G*, wildtype, rG4 mutant, and non-rG4 mutants of mirtrons (*D*) hsa-mir-877, (*E*) hsa-mir-1229, (*F*) hsa-mir-6834, and (*G*) hsa-mir-1234 are shown with (*B*) nontransfected and (*C*) empty vector transfected controls. (Scale bar = 50 μm). rG4, RNA G-quadruplex.

To understand how the rG4s in the 5′ arm of mirtrons contribute to splicing, we cloned the entire mirtron sequences of hsa-miR-877, hsa-miR-1229, hsa-miR-6834, and hsa-miR-1234 in the pEGFP-Mirt vector (Addgene #58330; containing a CMV promoter and an intron insertion site situated within the eGFP open reading frame) (23). The accurate splicing of the intron results in the in-frame fusion of the N and C termini of the eGFP encoding mRNA, facilitating the synthesis and expression of the eGFP protein (Fig. 5*A*). To evaluate the functionality of the pEGFP-Mirt vector system, we cloned two no-rG4-containing mirtrons (hsa-mir-6841 and hsa-mir-6888) into the pEGFP-Mirt vector and assessed their activity using flow cytometry and qRT-PCR (Fig. S2, *B–K*). In addition to rG4 mutants containing mirtrons, we also cloned non-rG4 mutants of the four mirtrons in the pEGFP-Mirt vector. The non-rG4 mutants (where mutations were introduced into the mirtrons without disrupting the rG4 or the stem-loop) were included as a control to assess the impact of mutations outside the rG4 on mirtron processing. The sequences of wildtype, rG4 mutant, and non-rG4 mutant of mirtrons are illustrated in Fig. 4. The constructs were transfected into HCT116 wildtype cells. A cleavage-independent role for Drosha in alternative splicing has been reported (69). Therefore, in addition to the HCT116 wildtype cells, we also used HCT116 *DROSHA* knockout cells to rule out an interplay between cleavage-independent functions of Drosha and rG4-mediated processing of mirtrons. Twenty-four hours posttransfection, the cells were assessed using fluorescence microscopy to evaluate eGFP expression, serving as a reporter for appropriate splicing, which leads to the maturation of mirtrons. The red-fluorescent signals from a cotransfected mCherry vector (Addgene #128744) acted as internal controls for transfection.

The eGFP fluorescence in HCT116 wildtype cells transfected with wildtype mirtrons (intact rG4s) indicated successful mirtron excision by splicing, leading to the formation of mature eGFP mRNA. Interestingly, introducing the rG4 interfering mutations reduced the eGFP fluorescence, suggesting impaired mirtron splicing. In comparison, the non-rG4 mutant mirtrons displayed eGFP fluorescence comparable to that in those with the wildtype mirtrons, implying that these mutations (outside the rG4 in the mirtron) do not affect the splicing of the mirtrons (Fig. 5, *B*–*G*). Similar results were obtained when the experiments were conducted in HCT116 *DROSHA* knockout cells (Fig. S3, *A*–*F*).

**Figure 6:**
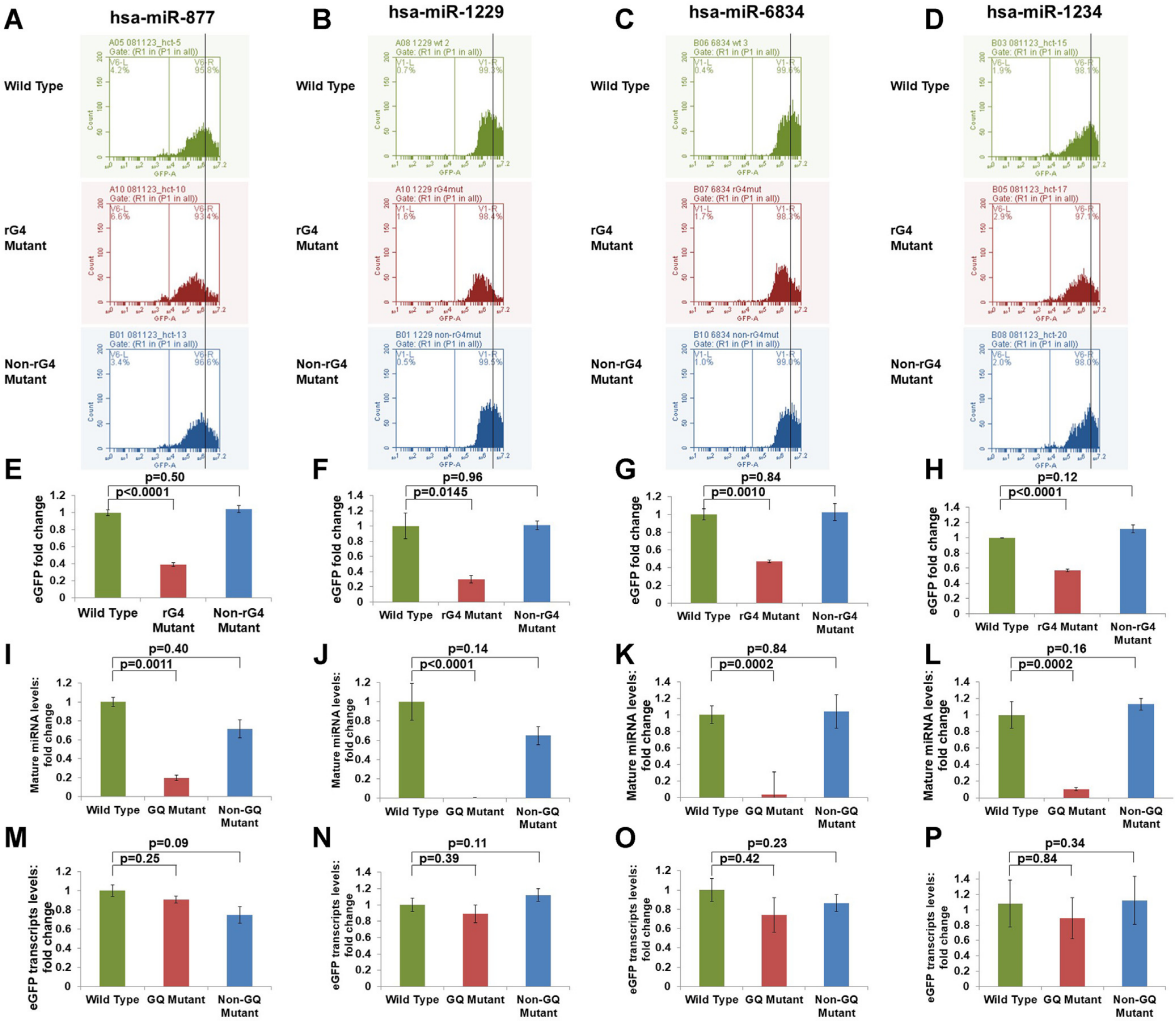
**Quantitative assessment of GFP-reporter splicing and mirtron biogenesis.***A–D*, flow cytometry analysis for GFP expression in mirtron wildtype, rG4 mutant, and non-rG4 mutant transfected in HCT116 wildtype cell line for (*A*) hsa-mir-877, (*B*) hsa-mir-1229, (*C*) hsa-mir-6834, and (*D*) hsa-mir-1234. *E*–*H*, bar graphs showing the median of GFP expression in mirtron wildtype, rG4 mutant, and non-rG4 mutant for (*E*) hsa-mir-877, (*F*) hsa-mir-1229, (*G*) hsa-mir-6834, and (*H*) hsa-mir-1234 transfected cells. *I*–*L*, differential mature miRNA expression in mirtron wildtype, rG4 mutant, and non-rG4 mutant for (*I*) hsa-mir-877-5′p, (*J*) hsa-mir-1229-5′p, (*K*) hsa-mir-6834-5′p, and (*L*) hsa-mir-1234-3′p. *M–P*, gene expression of eGFP transcripts in mirtron wildtype, rG4 mutant, and non-rG4 mutant for (*M*) hsa-mir-877, (*N*) hsa-mir-1229, (*O*) hsa-mir-6834, and (*P*) hsa-mir-1234. GQ, G-quadruplex; rG4, RNA G-quadruplex.

To quantitatively assess the effect of rG4s on splicing and mirtron generation, we also assessed the expression of the reporter eGFP fluorescence by flow cytometry. The eGFP fluorescence intensities from mCherry-positive cells were quantified for this purpose (Fig. S2*A*). Consistent with the microscopy data, HCT116 wildtype cells transfected with all four wildtype mirtrons demonstrated significantly higher eGFP expression levels compared to the respective rG4 mutant-transfected cells, while non-rG4 mutants did not affect the reporter eGFP expression (Fig. 6, *A*–*H*). Similar results were obtained for the wildtype mirtrons, rG4 mutant mirtrons, and non-rG4 mutant mirtrons in HCT116 *DROSHA*-knockout cells (Fig. S4, *A*–*H*). Taken together, these findings (i) suggest a key role for rG4s in the splicing of mirtrons and (ii) rule out a cleavage-independent role for Drosha in the rG4-mediated modulation of mirtron splicing.

To quantify the effect of rG4 on splicing, we also performed qRT-PCR to assess the proportion of unspliced transcript levels. We calculated the fold change for unspliced transcripts normalized to total transcript levels (the total transcript levels for the wildtype, rG4 mutant, and the non-rG4 mutant were comparable) using the delta-delta Ct method. We found that for rG4 mutants of all four mirtron (hsa-mir-877, hsa-mir-1229, hsa-mir-6834, and hsa-mir-1234), the unspliced eGFP transcript levels (normalized to total eGFP transcript levels) were significantly higher compared to the respective wildtype mirtron and the non-rG4 mutant mirtron in both HCT116 wildtype and HCT116 *DROSHA* knockout cells (Fig. S5). These findings confirm that the disruption of the rG4 structure (in rG4 mutants) specifically impairs the splicing efficiency of mirtrons, leading to a significantly higher proportion of unspliced transcripts as compared to the wildtype or the non-rG4 mutant mirtrons. These findings support a structural role for rG4s in facilitating splicing of mirtrons.

### rG4s in mirtrons impact the mirtrons-derived mature miRNA levels

To directly investigate the role of rG4s in the noncanonical maturation of miRNAs derived from mirtrons, we quantified the expression levels of mature miRNAs in HCT116 wildtype cells transfected with the wildtype, rG4 mutant, or the non-rG4-mutant reporter constructs. Expression levels of mature miRNAs were quantified using qRT-PCR using stem-loop primers. Consistent with the observations from fluorescence microscopy and flow cytometry analysis, cells with wildtype mirtrons had significantly higher levels of mature mirtron-derived miRNAs (Fig. 6, *I*–*L*). The introduction of rG4 disrupting mutations within the mirtron sequences resulted in a notable reduction and in some cases complete abrogation, of mature miRNA levels. The non-rG4 mutants were associated with mature miRNA levels comparable with that of the wildtype mirtrons (Fig. 6, *I*–*L*). Similar results were obtained with HCT116 *DROSHA* knockout cells (Fig. S4, *I*–*L*). As a control, we also monitored the total eGFP transcript levels (spliced and nonspliced) in the reporter-transfected cells. Interestingly, there were no significant differences in the total transcript levels between the wildtype, rG4 mutant, and non-rG4 mutant constructs for the four mirtrons studied in HCT116 wildtype and HCT116 *DROSHA* knockout cells (Figs. 6, *M*–*P* and S4, *M*–*P*). This clearly indicates that the differences observed in the mature miRNA levels are linked to posttranscriptional processing events. In sum, these findings highlight a previously unrecognized role for the rG4s at the 5′ arm of mirtrons in the splicing-dependent biogenesis of mirtrons.

## Discussion

Understanding the regulation of miRNA biogenesis remains an area of active research. Mirtrons are intron-derived miRNA precursors generated by splicing, independent of Drosha processing. The factors regulating splicing in the generation of mirtrons remain poorly understood. Here, we identify the abundance of rG4s at the 5′ arm of mirtrons and the widespread role of these RNA secondary structures in the processing of mirtrons.

We identified that rG4s are more abundant in mirtrons than in canonical pre-miRNAs. Remarkably, most rG4s in mirtrons are concentrated at the 5′ arm, while they are uniformly distributed along the length of canonical pre-miRNAs. As a result, more than 50% of human mirtrons have rG4s at the 5′ arm compared to only about 11% of canonical pre-miRNAs (Fig. 1*C*). In addition, nearly two-thirds of the rG4s in mirtrons were disrupted during subsequent processing, suggesting a potential role for these secondary structures in the biogenesis of mirtron-derived miRNA.

Mirtrons are much more abundant in vertebrates than in invertebrates or plants (70). It is well-documented that mirtrons/mirtron-derived miRNAs are less conserved between vertebrates and invertebrates than their canonical counterpart, indicating a more rapid evolution of mirtrons (71). Analysis of rG4s in mirtrons from different species indicates a particularly pronounced enrichment of rG4s among vertebrates, especially among mammals (Fig. 2). Interestingly, a recent report suggests that GQ numbers and densities increase with the organismal complexity of the species, with Aves and mammals having the highest proportion of genes with a GQ (72). Our analysis indicates that vertebrates have the highest proportion of mirtrons bearing a rG4, in keeping with their high GQ densities.

We chose four mirtrons with rG4s in the 5′ arm (*i.e.* one mirtron with a 2-tetrad GQ, two mirtrons with a 3-tetrad GQ, and one mirtron with a 4-tetrad GQ) to understand the functional role of rG4s in mirtrons. Biophysical characterization of RNA oligonucleotides corresponding to the predicted rG4s indicates that they form GQ structures *in vitro* and are stabilized by the GQ ligand BRACO-19.

We then studied the excision of mirtrons by splicing in the four mirtrons (hsa-mir-877, hsa-mir1229, hsa-mir-6834, and hsa-mir-1234) with a rG4 in the 5′ arm (wildtype mirtrons) using a GFP reporter assay in HCT116 wildtype and HCT116 *DROSHA* knockout cell lines. In parallel, we studied rG4 mutants (where the rG4 formation is disrupted by mutations without impacting the stem-loop) and non-rG4 mutants (where mutations that do not disrupt the rG4 are introduced without impacting the stem-loop). Our results provide several lines of evidence (eGFP levels by fluorescence microscopy, eGFP levels by FACS, and real-time PCR levels of mirtron-derived mature miRNAs) supporting a definitive role for the rG4s at the 5′ arm in the splicing of mirtrons. The splicing-mediated excision of mirtrons was significantly inhibited in the rG4 mutant mirtrons, leading to marked reduction or complete abrogation of mirtron-derived mature miRNA levels. While the extent of splicing and mature miRNA levels in the non-rG4 mutant mirtrons were comparable to that in the wildtype (Fig. 6, *I*–*L*), the eGFP mRNA levels (which are not affected by splicing) were comparable for the wildtype, rG4 mutant, and non-rG4 mutant-mirtron constructs, suggesting that the differences in the eGFP protein levels are due to regulation at the posttranscriptional level. This finding also rules out a potential role for DNA GQs in regulating the transcription of mirtrons.

Mirtrons represent splice-derived substrates for noncanonical miRNA biogenesis. A recent study identified structured splicing-derived RNAs (ssdRNAs) which have a stem-loop structure and represent a broad range of pre-miRNA mimics (73). The ssdRNAs/mirtrons may represent substrates that have access to the miRNA biogenesis machinery and may adversely impact the processing of canonical miRNA substrates and gene regulation (73). Thus, the cell must minimize the processing of splicing-derived pre-miRNA mimics (*i*.*e*., ssdRNAs/mirtrons) by the miRNA biogenesis machinery to maintain efficient processing of canonical miRNAs. Recent reports have identified two independent mechanisms that minimize the processing of ssdRNAs/mirtrons by canonical miRNA machinery in the cell: (a) 3′ tailing by terminal uridyl transferases (TUTases) and (b) a structural role of the DExD/H helicase domain of mammalian Dicer for high-fidelity miRNA biogenesis (73, 74). It has been documented that dominant mirtron-derived mature miRNA originates from the 3′ of the stem for invertebrates and from the 5′ of the stem for vertebrates (70). Evidence of 3′ tailing of mirtrons by TUTases is well documented among invertebrates. Our findings indicate that the enrichment of rG4s in the 5′ arm of mirtrons may represent an additional layer of control to regulate the splicing-derived pre-miRNA mimics generated independently of Drosha processing among vertebrates.

Splicing is regulated by multiple RNA-binding proteins [including heterogeneous nuclear ribonucleoproteins]. Previous studies have demonstrated a role for rG4s in regulating splicing through modulation of these RNA-binding proteins (49, 66). While the link between splicing and rG4s has been previously described, the role of rG4s in the 5′ arm of mirtrons in the modulation of splicing of mirtrons has not been previously recognized. However, unlike canonical pre-mRNA splicing, the splicing of mirtrons may be impacted by the dynamics of rG4-and hairpin-formation. Our results extend this understanding by highlighting the critical contribution of rG4 formation in mediating splicing events crucial for mirtron-mediated, Drosha-independent miRNA biogenesis.

Furthermore, rG4s are enriched at splice sites, particularly in Aves and mammals (66); this is in keeping with our findings that the 5′ arm of mirtrons in chicken and mammals are enriched for rG4s. However, GQs are enriched at both the 5′ and the 3′ splice sites of introns (65), suggesting that the selective enrichment of rG4s at the 5′ arm of mirtrons may not represent a general enrichment of these secondary structures at splice sites.

Our findings unveil how rG4s influence mirtron biology and their potential role as an additional layer of control to minimize the availability of splicing-derived mirtrons for subsequent processing by the cytoplasmic miRNA processing machinery (*i.e.*, DICER-mediated cleavage and Argonaute proteins). This work lays the groundwork for a plethora of studies to further our understanding of mechanisms regulating mirtrons/splicing-derived pre-miRNA mimics from contaminating the canonical miRNA biogenesis pathways.

## Conclusion

Mirtrons represent an important class of noncanonical miRNAs that are generated by splicing without the need for Drosha-dependent processing in the nucleus. The need to minimize Dicer/Ago-mediated cytoplasmic processing of splicing-derived hairpins/mirtrons (*i.e.*, pre-miRNA mimics) is increasingly recognized as an essential step to allow efficient processing of canonical miRNA. The 3′ tailing of mirtron hairpins by TUTases is reported to suppress the biogenesis of mirtrons among invertebrates. The mechanisms regulating mirtron biogenesis among vertebrates are not well understood. GQs are nucleic acid secondary structures (present in both DNA and RNA) that regulate a plethora of cellular processes. On careful analysis of mirtrons, we identified the presence of an rG4 in the majority (over 50%) of the 5′ arm of mirtrons from mammalian mirtrons but not from invertebrate or plant mirtrons. We also demonstrate that the predicted rG4s form a GQ structure *in vitro* for a subset of human mirtrons. Furthermore, using flow cytometry, fluorescence microscopy, and real-time PCR, we show that the rG4 at the 5′ arm facilitates the splicing of mirtrons. Importantly, mutations disrupting the rG4 at the 5′ arm but not other mutations in the mirtron led to significant inhibition of splicing. Furthermore, mutations disrupting the rG4 in the 5′ arm led to inhibition or complete abrogation of miRNA maturation. This effect of rG4s on splicing of mirtrons was found to be comparable in HCT116 wildtype and HCT116 *DROSHA* knockout cell lines. Our findings identify rG4s in the 5′ arm as key regulators of mammalian mirtron biogenesis. This work highlights how rG4s exert an additional layer of control of noncanonical miRNA biogenesis among mammals.

## Experimental procedures

### Mining of rG4s in mirtron and canonical pre-miRNA

Mirtron and canonical pre-miRNA sequences were downloaded from miRBase (v22.1) (https://mirbase.org/) (75). The QGRS mapper software (https://bioinformatics.ramapo.edu/QGRS/index.php) (76) was used to search for the presence and position of putative rG4s with an iteration of a minimum of two guanine residues repeated at least four times with loop lengths ranging from one to seven residues {(G≥2X1-7)}4 (77). The rG4 density was then calculated as the number of nonoverlapping putative rG4s per hundred bases of the pre-miRNA length (73).

### Randomization of sequences

To assess whether the presence of rG4 motifs in mirtron sequences is a result of a random or nonrandom process, the human mirtron sequences underwent shuffling while maintaining dinucleotide frequencies. This involved a dinucleotide shuffle of the selected sequences, preserving the overall nucleotide composition. The shuffling process, repeated four times, utilized an online tool “Sequence Manipulation Suite” (https://www.bioinformatics.org/sms2/) (78). The resulting randomized sequences were analyzed for rG4s, which were then compared to those in the original sequences.

### Evolutionary analysis

Validated mirtron sequences from various organisms were downloaded from MirtronDB (http://mirtrondb.cp.utfpr.edu.br/index.php) (79). The sequences were analyzed for the presence and the position of rG4 with a similar iteration of {(G≥2X1-7)}4. The organisms containing at least five mirtron sequences (n ≥ 5) were analyzed further for 5′ enrichment of rG4s.

The first 25 nucleotides (5′ end) of all the mirtrons from different taxonomies: plants, invertebrates, and vertebrates were downloaded from MirtronDB. The webLogo 3 software (https://weblogo.threeplusone.com/create.cgi) (80) was used to align the sequences and create the logo indicating the probability of each nucleotide at every position in the first 25 nucleotides of mirtron sequences analyzed.

### Selection of rG4-containing mirtrons for functional studies

Multimeric GQs often interact with other local GQs, and the methods to study their structure and function are still evolving (81). For experimental studies, we selected four mirtrons with a single rG4 (one 2-tetrad, two 3-tetrad, and one 4-tetrad rG4s) motif so that our findings can be directly related to all types of rG4 motif. The sequences of the RNA oligonucleotides of wildtype and rG4 mutant are mentioned in Table S2.

### CD spectroscopy

CD studies were performed on Jasco 815. RNA oligonucleotides were purchased from Genscript for all biophysical experiments. RNA oligonucleotides were prepared in a final concentration of 5 μM with 10 mM sodium cacodylate buffer (pH −7.4) and 50 mM potassium chloride (KCl). The samples were slowly cooled to room temperature after heating at 95 °C for 5 min. Spectra at 220 to 320 nm wavelength range were recorded in a quartz cuvette of 1 mm path length, keeping a 1 nm step size and time of 1s per point. A buffer baseline was recorded and subtracted from the sample spectra.

CD melt curves were obtained over 20 to 95 °C using the same oligonucleotide concentration (5 μM), either without or with 10 μM BRACO19 as previously described by Kumar *et al.* (82). Melting temperatures (T_m_) were calculated by fitting the curve into the Boltzmann function with Origin 9.7.5 (Origin Lab Corp).

### UV melting analysis

A Cary 100 Bio UV-Vis double-beam spectrophotometer (Agilent Technologies) equipped with a multicell holder attached to a Peltier controller was used to perform UV melting experiments. RNA oligonucleotides at a concentration of 4 μM were mixed with 10 mM sodium cacodylate (pH −7.5) and 100 mM KCl. The melting curves were recorded at 295 nm between 20 °C and 95 °C with a ramp rate of 1 °C/min as previously described by Kumar *et al.* (81). Origin 9.7.5 (Origin Lab Corp) was used to analyze and plot melting curves.

### Mirtron constructs

The mirtron forward and reserve complementary sequence was commercially synthesized by Eurofins listed in Table S3. The complementary oligonucleotides were mixed in 1:1 M ratio to anneal in annealing buffer [10 mM Tris, 1 mM EDTA, and 50 mM NaCl (pH = 8)] by slowly cooling to room temperature after heating at 95 °C for 5 min. The wildtype and mutant annealed sequences were cloned in pEGFP-Mirt vector (Addgene) using the protocol Sibley *et al.* (23, 83). The plasmid constructs were extracted using QIAprep Spin Miniprep Kit (Qiagen) and confirmed by sequencing.

### Fluorescence microscopy

HCT116 wildtype and *DROSHA* knockout cells (procured from KCTC) were maintained in McCoy's medium (Invitrogen) supplemented with 10% fetal bovine serum and were incubated at 37 °C and with 5% CO_2_. Cells were seeded in 24-well plate at a density of 1 × 10^5^ cells/well. The mirtron constructs (wildtype or mutant; 300 ng each) and 350 ng of pcDNA3.1-mCherry (transfection control) were cotransfected using Lipofectamine 2000. At 24 h posttransfection, cells were visualized under fluorescence microscopy for GFP and mCherry expression.

### Flow cytometry

HCT116 wildtype and *DROSHA* knockout cells transfected with the mirtron constructs pcDNA3.1-mCherry (transfection control) were trypsinized after 24 h and centrifuged at 1000 rpm for 5 min. The cell pellets were resuspended in phosphate buffer saline with 1% fetal bovine serum and 2 mM EDTA. The GFP and mCherry fluorescence were acquired using BD Accuri C6 flow-cytometer, and data were processed using Cflow software. MCherry-positive cells were gated as transfected cells and were monitored for GFP fluorescence. The median GFP fluorescence intensities were calculated. The experiments were done in triplicates, and mean ± SD were plotted.

### Differential gene expression

HCT116 wildtype and *DROSHA* knockout cells transfected with the mirtron constructs pcDNA3.1-mCherry (transfection control) used for total RNA were extracted using the standard TRIzol reagent protocol. RNA concentrations were measured using Implen Nanophotometer N60. cDNA synthesis was carried out using the cDNA synthesis kit (Edna) with 1 μg of DNase I-treated RNA. The stem-loop method was used to prepare miRNA-specific cDNA (84). qPCR was then performed in triplicates using the Takara Universal SYBR Green (85). The stem-loop primers for cDNA synthesis and qPCR primers are mentioned in Table S4.

### Data analyses

Data were plotted as mean values ± SD from at least three experiments. Statistical significance was defined as *p* < 0.05 calculated using Student's *t* test unless stated otherwise. Origin 9.7.5 (Origin Lab Corp) was used for violin plots and melt curves. Microsoft Excel 365 was used to plot bar graphs. CFlow software was used for flow cytometry analysis.

## Data availability

This paper does not report any original code. All data reported in this paper will be shared by the lead contact upon request.

## Supporting information

This article contains supporting information.

## Conflict of interests

The authors declare no conflict of interest with the contents of this article.

## References

[1] Saliminejad K., Khorram Khorshid H.R., Soleymani Fard S., Ghaffari S.H. (2019). An overview of microRNAs: biology, functions, therapeutics, and analysis methods. J. Cell Physiol..

[2] Mohr A.M., Mott J.L. (2015). Overview of microRNA biology. Semin. Liver Dis..

[3] Ho P.T.B., Clark I.M., Le L.T.T. (2022). MicroRNA-based diagnosis and therapy. Int. J. Mol. Sci..

[4] Diener C., Keller A., Meese E. (2022). Emerging concepts of miRNA therapeutics: from cells to clinic. Trends Genet..

[5] Lee Y., Kim M., Han J., Yeom K.H., Lee S., Baek S.H. (2004). MicroRNA genes are transcribed by RNA polymerase II. EMBO J..

[6] Borchert G.M., Lanier W., Davidson B.L. (2006). RNA polymerase III transcribes human microRNAs. Nat. Struct. Mol. Biol..

[7] Han J., Lee Y., Yeom K.H., Kim Y.K., Jin H., Kim V.N. (2004). The Drosha-DGCR8 complex in primary microRNA processing. Genes Dev..

[8] Lee Y., Jeon K., Lee J.T., Kim S., Kim V.N. (2002). MicroRNA maturation: stepwise processing and subcellular localization. EMBO J..

[9] Yeom K.H., Lee Y., Han J., Suh M.R., Kim V.N. (2006). Characterization of DGCR8/Pasha, the essential cofactor for Drosha in primary miRNA processing. Nucleic Acids Res..

[10] Yi R., Qin Y., Macara I.G., Cullen B.R. (2003). Exportin-5 mediates the nuclear export of pre-microRNAs and short hairpin RNAs. Genes Dev..

[11] Zeng Y., Cullen B.R. (2004). Structural requirements for pre-microRNA binding and nuclear export by Exportin 5. Nucleic Acids Res..

[12] Bernstein E., Caudy A.A., Hammond S.M., Hannon G.J. (2001). Role for a bidentate ribonuclease in the initiation step of RNA interference. Nature.

[13] Khvorova A., Reynolds A., Jayasena S.D. (2003). Functional siRNAs and miRNAs exhibit strand bias. Cell.

[14] Lin S.L., Chang D., Ying S.Y. (2005). Asymmetry of intronic pre-miRNA structures in functional RISC assembly. Gene.

[15] Schwarz D.S., Hutvágner G., Du T., Xu Z., Aronin N., Zamore P.D. (2003). Asymmetry in the assembly of the RNAi enzyme complex. Cell.

[16] Baek D., Villén J., Shin C., Camargo F.D., Gygi S.P., Bartel D.P. (2008). The impact of microRNAs on protein output. Nature.

[17] Zeng Y., Yi R., Cullen B.R. (2003). MicroRNAs and small interfering RNAs can inhibit mRNA expression by similar mechanisms. Proc. Natl. Acad. Sci. U. S. A..

[18] Ruby J.G., Jan C.H., Bartel D.P. (2007). Intronic microRNA precursors that bypass Drosha processing. Nature.

[19] Salim U., Kumar A., Kulshreshtha R., Vivekanandan P. (2022). Biogenesis, characterization, and functions of mirtrons. Wiley Inter. Rev. RNA.

[20] Okamura K., Hagen J.W., Duan H., Tyler D.M., Lai E.C. (2007). The mirtron pathway generates microRNA-class regulatory RNAs in Drosophila. Cell.

[21] Flynt A.S., Greimann J.C., Chung W.J., Lima C.D., Lai E.C. (2010). MicroRNA biogenesis *via* splicing and exosome-mediated trimming in Drosophila. Mol. Cell.

[22] Menzel P., Mccorkindale A.L., Stefanov S.R., Zinzen R.P., Meyer I.M. (2019). Transcriptional dynamics of microRNAs and their targets during Drosophila neurogenesis. RNA Biol..

[23] Seow Y., Sibley C.R., Wood M.J.A. (2012). Artificial mirtron-mediated gene knockdown: functional DMPK silencing in mammalian cells. RNA.

[24] Amourda C., Saunders T.E. (2020). The mirtron miR-1010 functions in concert with its host gene SKIP to balance elevation of nAcR β 2. Sci. Rep..

[25] Zhang W.G., Chen L., Dong Q., He J., Zhao H.D., Li F.L. (2013). Mmu-miR-702 functions as an anti-apoptotic mirtron by mediating ATF6 inhibition in mice. Gene.

[26] Sakai E., Miura Y., Suzuki-Kouyama E., Oka K., Tachibana M., Kawabata K. (2017). A mammalian mirtron MIR-1224 promotes tube-formation of human primary endothelial cells by targeting anti-angiogenic factor epsin2. Sci. Rep..

[27] Baghdadi M.B., Firmino J., Soni K., Evano B., Di Girolamo D., Mourikis P. (2018). Notch-induced miR-708 antagonizes satellite cell migration and maintains quiescence. Cell Stem Cell.

[28] Butkytė S., Čiupas L., Jakubauskienė E., Vilys L., Mocevicius P., Kanopka A. (2016). Splicing-dependent expression of microRNAs of mirtron origin in human digestive and excretory system cancer cells. Clin Epigenet..

[29] Zhao M., Hou Y., Du Y.E., Yang L., Qin Y., Peng M. (2020). Drosha-independent miR-6778–5p strengthens gastric cancer stem cell stemness *via* regulation of cytosolic one-carbon folate metabolism. Cancer Lett..

[30] An J.X., Ma M.H., Zhang C.D., Shao S., Zhou N.M., Dai D.Q. (2018). miR-1236-3p inhibits invasion and metastasis in gastric cancer by targeting MTA2. Cancer Cell Int..

[31] Sibley C.R., Seow Y., Curtis H., Weinberg M.S., Wood M.J.A. (2012). Silencing of Parkinson's disease-associated genes with artificial mirtron mimics of miR-1224. Nucleic Acids Res..

[32] Kock K.H., Kong K.W., Hoon S., Seow Y. (2015). Functional VEGFA knockdown with artificial 3′-tailed mirtrons defined by 5′ splice site and branch point. Nucleic Acids Res..

[33] Curtis H.J., Seow Y., Wood M.J.A., Varela M.A. (2017). Knockdown and replacement therapy mediated by artificial mirtrons in spinocerebellar ataxia 7. Nucleic Acids Res..

[34] Huppert J.L., Balasubramanian S. (2005). Prevalence of quadruplexes in the human genome. Nucleic Acids Res..

[35] Huppert J.L., Balasubramanian S. (2007). G-quadruplexes in promoters throughout the human genome. Nucleic Acids Res..

[36] Bochman M.L., Paeschke K., Zakian V.A. (2012). DNA secondary structures: stability and function of G-quadruplex structures. Nat. Rev. Genet..

[37] Patel D.J., Phan A.T., Kuryavyi V. (2007). Human telomere, oncogenic promoter and 5′-UTR G-quadruplexes: diverse higher order DNA and RNA targets for cancer therapeutics. Nucleic Acids Res..

[38] Ray S., Bandaria J.N., Qureshi M.H., Yildiz A., Balci H. (2014). G-quadruplex formation in telomeres enhances POT1/TPP1 protection against RPA binding. Proc. Natl. Acad. Sci. U. S. A..

[39] Valton A.L., Prioleau M.N. (2016). G-quadruplexes in DNA replication: a problem or a necessity?. Trends Genet..

[40] Kumari S., Bugaut A., Balasubramanian S. (2008). Position and stability are determining factors for translation repression by an RNA G-quadruplex-forming sequence within the 5′ UTR of the NRAS proto-oncogene. Biochemistry.

[41] Arora A., Suess B. (2011). An RNA G-quadruplex in the 3′ UTR of the proto-oncogene PIM1 represses translation. RNA Biol..

[42] Arora A., Dutkiewicz M., Scaria V., Hariharan M., Maiti S., Kurreck J. (2008). Inhibition of translation in living eukaryotic cells by an RNA G-quadruplex motif. RNA.

[43] Robinson J., Raguseo F., Nuccio S.P., Liano D., Di Antonio M. (2021). DNA G-quadruplex structures: more than simple roadblocks to transcription?. Nucleic Acids Res..

[44] Zhang J.Y., Xia Y., Hao Y.H., Tan Z. (2020). DNA:RNA hybrid G-quadruplex formation upstream of transcription start site. Sci. Rep..

[45] Shen J., Varshney D., Simeone A., Zhang X., Adhikari S., Tannahill D. (2021). Promoter G-quadruplex folding precedes transcription and is controlled by chromatin. Genome Biol..

[46] Endoh T., Kawasaki Y., Sugimoto N. (2013). Translational halt during elongation caused by G-quadruplex formed by mRNA. Methods..

[47] Bugaut A., Balasubramanian S. (2012). 5′-UTR RNA G-quadruplexes: translation regulation and targeting. Nucleic Acids Res..

[48] Kumari S., Bugaut A., Huppert J.L., Balasubramanian S. (2007). An RNA G-quadruplex in the 5′ UTR of the NRAS proto-oncogene modulates translation. Nat. Chem. Biol..

[49] Huang H., Zhang J., Harvey S.E., Hu X., Cheng C. (2017). RNA G-quadruplex secondary structure promotes alternative splicing *via* the RNA-binding protein hnRNPF. Genes Dev..

[50] Neckles C., Boer R.E., Aboreden N., Cross A.M., Walker R.L., Kim B.H. (2019). HNRNPH1-dependent splicing of a fusion oncogene reveals a targetable RNA G-quadruplex interaction. RNA.

[51] Didiot M.C., Tian Z., Schaeffer C., Subramanian M., Mandel J.L., Moine H. (2008). The G-quartet containing FMRP binding site in FMR1 mRNA is a potent exonic splicing enhancer. Nucleic Acids Res..

[52] Marcel V., Tran P.L.T., Sagne C., Martel-Planche G., Vaslin L., Teulade-Fichou M.P. (2011). G-quadruplex structures in TP53 intron 3: role in alternative splicing and in production of p53 mRNA isoforms. Carcinogenesis.

[53] Tran P.L.T., Mergny J.L., Alberti P. (2011). Stability of telomeric G-quadruplexes. Nucleic Acids Res..

[54] Nguyen G.H., Tang W., Robles A.I., Beyer R.P., Gray L.T., Welsh J.A. (2014). Regulation of gene expression by the BLM helicase correlates with the presence of G-quadruplex DNA motifs. Proc. Natl. Acad. Sci. U. S. A..

[55] Schiavone D., Guilbaud G., Murat P., Papadopoulou C., Sarkies P., Prioleau M. (2014). Determinants of G quadruplex-induced epigenetic instability in REV 1-deficient cells. EMBO J..

[56] Sengupta A., Roy S.S., Chowdhury S. (2021). Non-duplex G-quadruplex DNA structure: a developing story from predicted sequences to DNA structure-dependent epigenetics and beyond. Acc. Chem. Res..

[57] Sanchez-Martin V., Soriano M., Garcia-Salcedo J.A. (2021). Quadruplex ligands in cancer therapy. Cancers (Basel).

[58] Kosiol N., Juranek S., Brossart P., Heine A., Paeschke K. (2021). G-quadruplexes: a promising target for cancer therapy. Mol. Cancer.

[59] Santos T., Salgado G.F., Cabrita E.J., Cruz C. (2021). G-quadruplexes and their ligands: biophysical methods to unravel g-quadruplex/ligand interactions. Pharmaceuticals.

[60] Zhang S., Wu Y., Zhang W. (2014). G-quadruplex structures and their interaction diversity with ligands. ChemMedChem..

[61] Che T., Wang Y.Q., Huang Z.L., Tan J.H., Huang Z.S., Chen S.B. (2018). Natural alkaloids and heterocycles as G-quadruplex ligands and potential anticancer agents. Molecules.

[62] Burge S., Parkinson G.N., Hazel P., Todd A.K., Neidle S. (2006). Quadruplex DNA: sequence, topology and structure. Nucleic Acids Res..

[63] Dingley A.J., Peterson R.D., Grzesiek S., Feigon J. (2005). Characterization of the cation and temperature dependence of DNA quadruplex hydrogen bond properties using high-resolution NMR. J Am Chem Soc..

[64] Zaccaria F., Fonseca Guerra C. (2018). RNA *versus* DNA G-quadruplex: the origin of increased stability. Chemistry.

[65] Jara-Espejo M., Fleming A.M., Burrows C.J. (2020). Potential G-quadruplex forming sequences and N6-methyladenosine colocalize at human pre-mRNA intron splice sites. ACS Chem. Biol..

[66] Georgakopoulos-Soares I., Parada G.E., Wong H.Y., Medhi R., Furlan G., Munita R. (2022). Alternative splicing modulation by G-quadruplexes. Nat. Commun..

[67] Rasschaert P., Figueroa T., Dambrine G., Rasschaert D., Laurent S. (2016). Alternative splicing of a viral mirtron differentially affects the expression of other microRNAs from its cluster and of the host transcript. RNA Biol..

[68] Rouleau S.G., Garant J.M., Bolduc F., Bisaillon M., Perreault J.P. (2018). G-Quadruplexes influence pri-microRNA processing. RNA Biol..

[69] Havens M.A., Reich A.A., Hastings M.L. (2014). Drosha promotes splicing of a pre-microRNA-like alternative exon. PLoS Genet..

[70] Berezikov E., Chung W.J., Willis J., Cuppen E., Lai E.C. (2007). Mammalian mirtron genes. Mol. Cell.

[71] Axtell M.J., Westholm J.O., Lai E.C. (2011). Vive la différence: Biogenesis and evolution of microRNAs in plants and animals. Genome Biol..

[72] Wu F., Niu K., Cui Y., Li C., Lyu M., Ren Y. (2021). Genome-wide analysis of DNA G-quadruplex motifs across 37 species provides insights into G4 evolution. Commun. Biol..

[73] Lee S., Jee D., Srivastava S., Yang A., Ramidi A., Shang R. (2023). Promiscuous splicing-derived hairpins are dominant substrates of tailing-mediated defense of miRNA biogenesis in mammals. Cell Rep..

[74] Zapletal D., Taborska E., Pasulka J., Malik R., Kubicek K., Zanova M. (2022). Structural and functional basis of mammalian microRNA biogenesis by Dicer. Mol Cell.

[75] Kozomara A., Birgaoanu M., Griffiths-Jones S. (2019). MiRBase: from microRNA sequences to function. Nucleic Acids Res..

[76] Kikin O., D'Antonio L., Bagga P.S. (2006). QGRS Mapper: a web-based server for predicting G-quadruplexes in nucleotide sequences. Nucleic Acids Res..

[77] Pandey S., Agarwala P., Maiti S. (2013). Effect of loops and G-quartets on the stability of RNA G-quadruplexes. J. Phys. Chem. B.

[78] Stothard P. (2000). The sequence manipulation suite: JavaScript programs for analyzing and formatting protein and DNA sequences. Biotechniques.

[79] Da Fonseca B.H.R., Domingues D.S., Paschoal A.R. (2019). MirtronDB: a mirtron knowledge base. Bioinformatics.

[80] Crooks G.E., Hon G., Chandonia J.M., Brenner S.E. (2004). WebLogo: a sequence logo generator. Genome Res..

[81] Kumar S., Choudhary D., Patra A., Bhavesh N.S., Vivekanandan P. (2020). Analysis of G-quadruplexes upstream of herpesvirus miRNAs: evidence of G-quadruplex mediated regulation of KSHV miR-K12-1-9,11 cluster and HCMV miR-US33. BMC Mol. Cell Biol..

[82] Kumar A., Kamuju V., Vivekanandan P. (2024). RNA G-quadruplexes inhibit translation of the PE/PPE transcripts in Mycobacterium tuberculosis. J. Biol. Chem..

[83] Sibley C.R., Seow Y., Saayman S., Dijkstra K.K., El Andaloussi S., Weinberg M.S. (2012). The biogenesis and characterization of mammalian microRNAs of mirtron origin. Nucleic Acids Res..

[84] Schmittgen T.D., Lee E.J., Jiang J., Sarkar A., Yang L., Elton T.S. (2008). Real-time PCR quantification of precursor and mature microRNA. Methods.

[85] Lu T.X., Rothenberg M.E. (2018). MicroRNA. J. Allergy Clin. Immunol..

