# Supplementary material for: RNA G-quadruplexes regulate mammalian mirtron biogenesis
Source: J Biol Chem. 2025 Feb 7;301(3):108276. doi: 10.1016/j.jbc.2025.108276 (PMC11927685; doi:10.1016/j.jbc.2025.108276)
Supplement: Supporting Information [file mmc1.docx]

**RNA G-Quadruplexes regulate mammalian mirtron biogenesis**

Uzma Salim^1^, Manoj B. Menon^1^, Sonam Dhamija^2,3*^ and Perumal Vivekanandan^1*^

^1^Kusuma School of Biological Sciences, Indian Institute of Technology, Hauz Khas, New Delhi-110016, India

^2^CSIR-Institute of Genomics & Integrative Biology, South Campus, Mathura Road, Sukhdev Vihar, New Delhi – 110025, India

^3^Academy of Scientific and Innovative Research (AcSIR), Ghaziabad, India

*Corresponding Author(s)

Correspondence: [vperumal@bioschool.iitd.ac.in](mailto:vperumal@bioschool.iitd.ac.in)

[sonam.dhamija@igib.res.in](mailto:sonam.dhamija@igib.res.in)

**TABLE OF CONTENTS**

| S.No. | Content | Description | Page No. |
| --- | --- | --- | --- |
| 1. | Figure S1 | CD melting analyses of mirtron-derived rG4s | S2 |
| 2. | Figure S2 | Schematic representation and quantitative assessment of no-rG4 containing mirtrons | S3 |
| 3. | Figure S3 | GFP splicing reporter for mirtron biogenesis | S5 |
| 4. | Figure S4 | Quantitative assessment of GFP-reporter splicing and mirtron biogenesis | S6 |
| 5. | Figure S5 | Quantitative assessment of unspliced transcript levels | S7 |
| 6. | Table S1 | rG4-motif analyses of human mirtrons | S8 |
| 7. | Table S2 | RNA oligonucleotide sequences of selected human mirtron rG4s | S13 |
| 8. | Table S3 | Cloning primer sequences of selected human mirtron rG4s | S14 |
| 9. | Table S4 | qPCR primer sequences | S16 |

**SUPPORTING INFORMATION**

**Figure S1**

**Wild type**

**Wild type with Ligand**

**rG4 mutant**

**rG4 mutant with Ligand**

**AA**


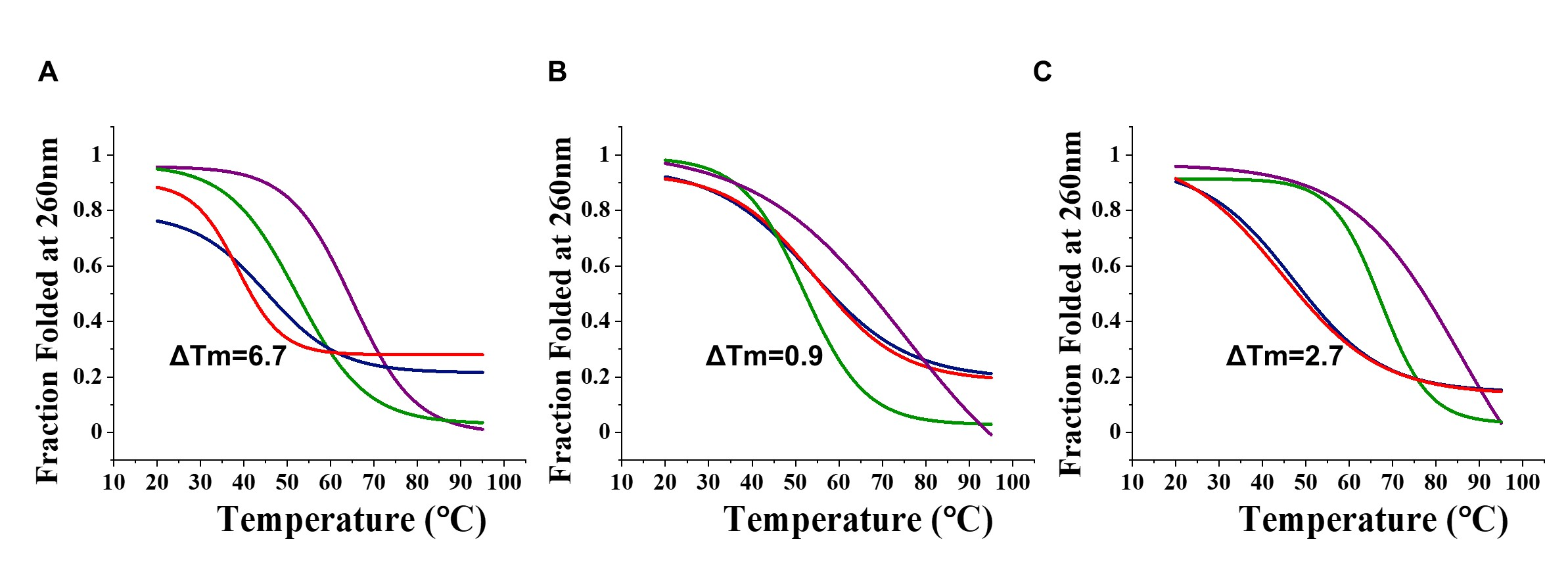


**Figure S1: CD melting analyses of mirtron-derived rG4s**. (A-C) CD melt curves showing BRACO19-mediated stabilization of the wildtype rG4 in the RNA oligonucleotides from mirtrons (A) hsa-mir-877, (B) hsa-mir-1229, and (C) hsa-mir-6834 while no change in melting temperature on rG4 mutant oligonucleotides on the addition of BRACO19.

**Figure S2**

A


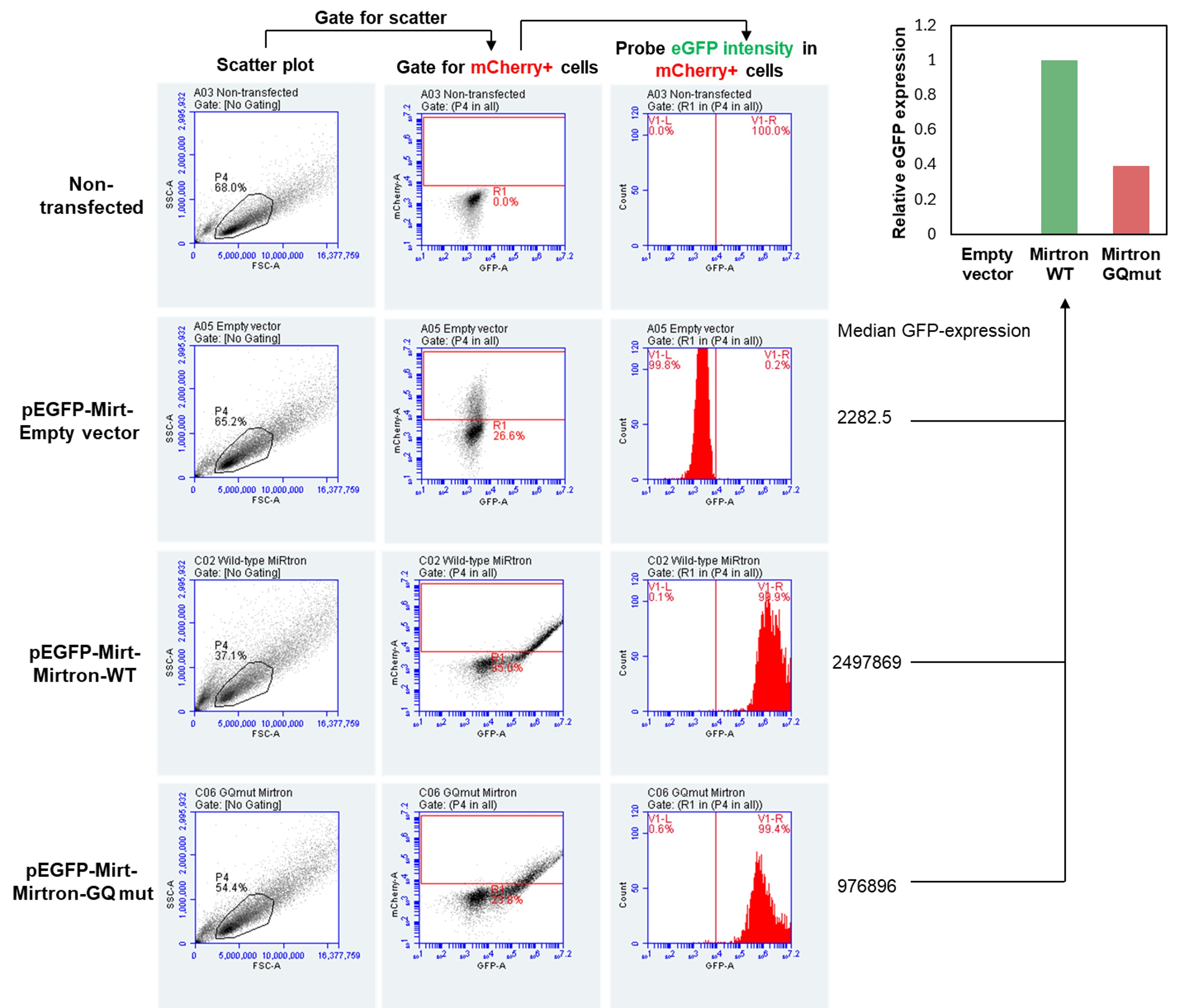


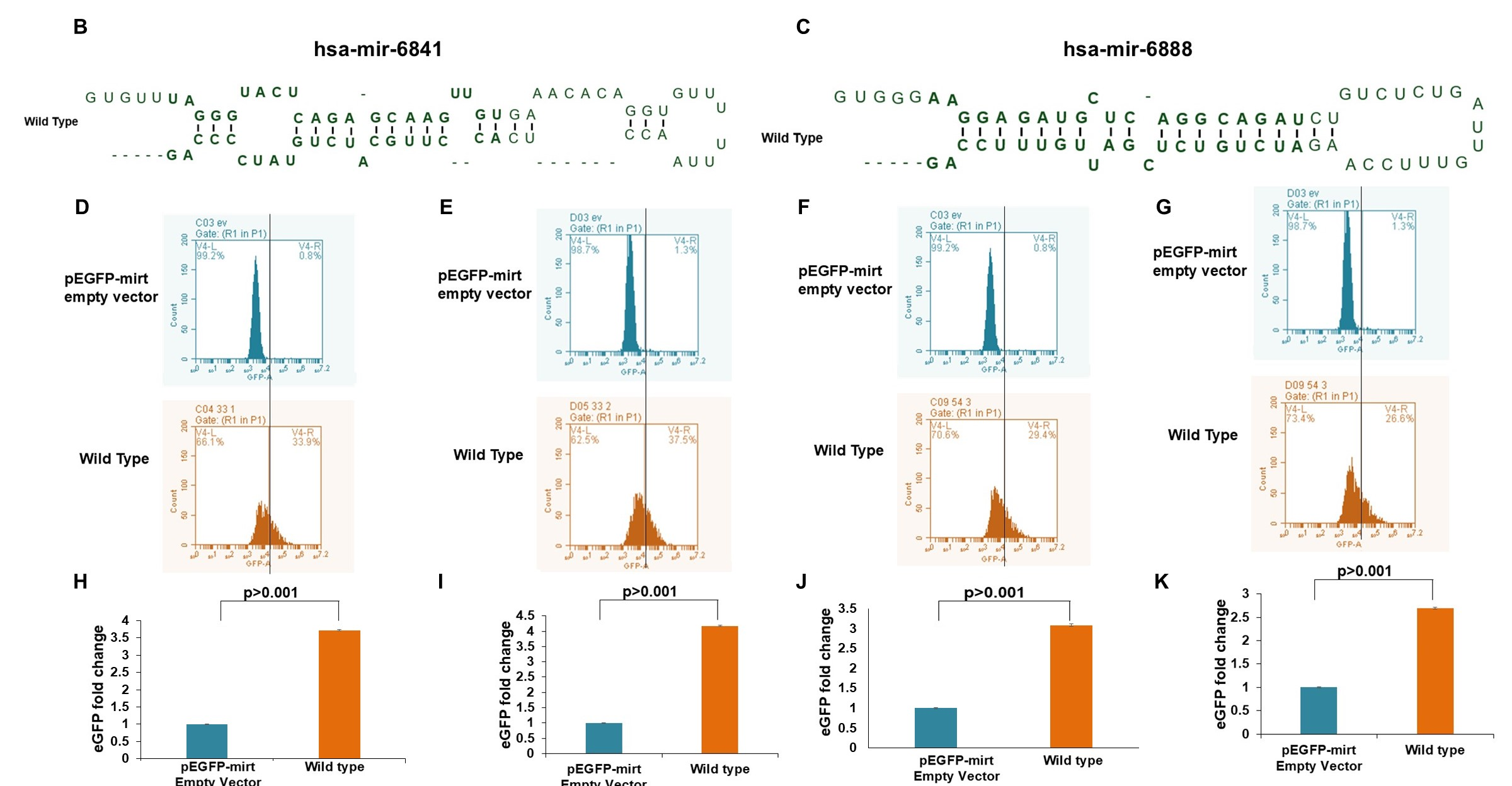


**Figure S2:** (A) Schematic representation of gating pattern employed for flow cytometry data shown in Figure 6. (B-K) Diagrammatic representation of stem-loop structures of mirtron (B) hsa-mir-6841 and (C) hsa-mir-6888. Flow cytometry analysis for GFP expression in mirtron wild type hsa-mir-6841 transfected in (D) HCT116 wild type and (E) HCT116 *DROSHA* knockout cell line and mirtron wildtype hsa-mir-6888 transfected in (F) HCT116 wild type and (G) HCT116 *DROSHA* knockout cell line. Bar graphs showing the median of GFP expression in mirtron wildtype hsa-mir-6841 transfected in (H) HCT116 wildtype and (I) HCT116 *DROSHA* knockout cell line and mirtron wildtype hsa-mir-6888 transfected in (J) HCT116 wildtype and (K) HCT116 *DROSHA* knockout cell line.

**Figure S3**


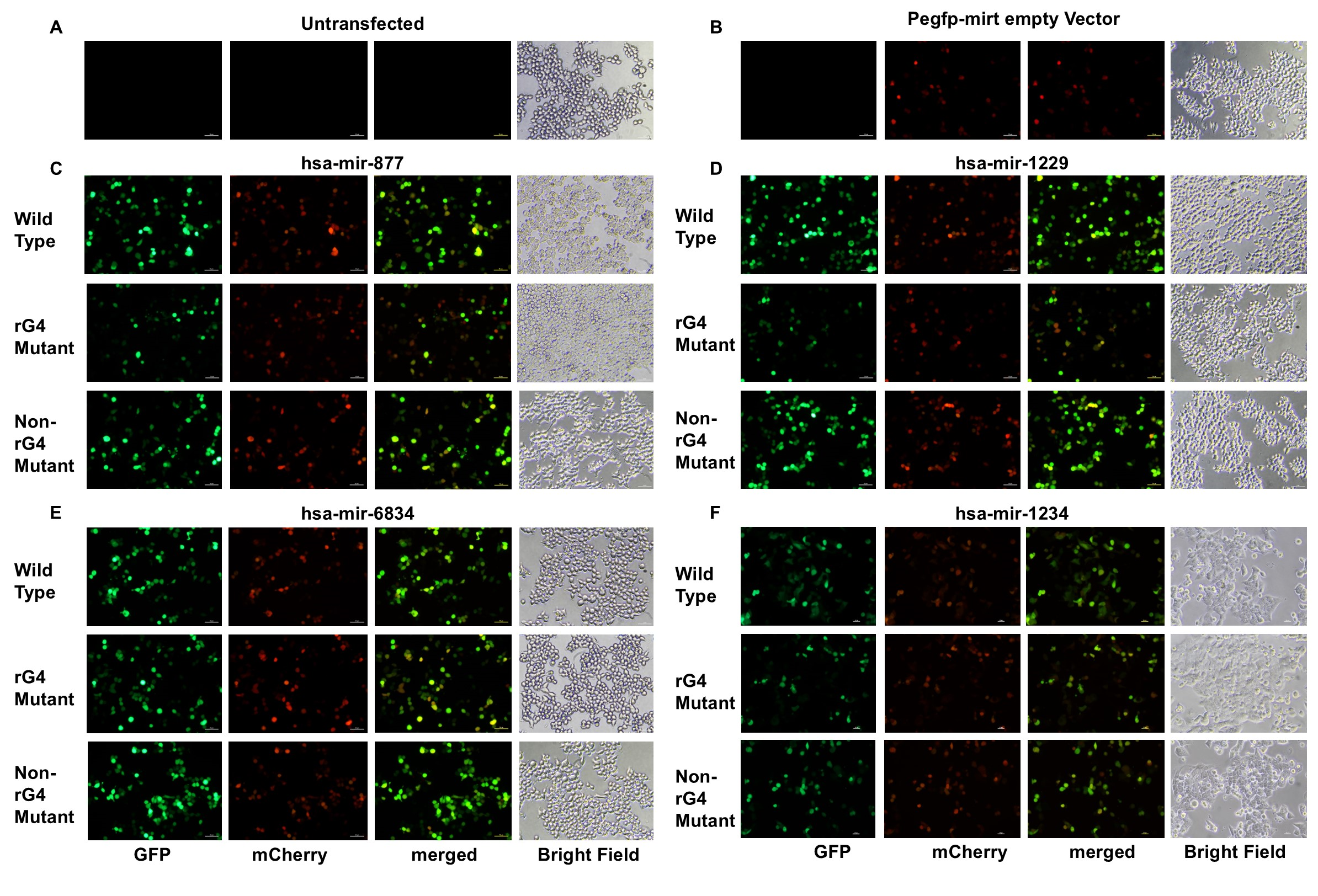


**Figure S3:** Fluorescence Microscopy for GFP expression on appropriate splicing of mirtrons from the intronic region between artificial exons encoding the N- and C-terminal regions of eGFP in peGFP-mirt vector transfected in HCT116 *DROSHA* knockout cell line. Red fluorescence from co-transfected pcDNA-mCherry is shown as an internal control. Wildtype, rG4 mutant and non-rG4 mutants of mirtrons (C) hsa-mir-877, (D) hsa-mir-1229, (E) hsa-mir-6834 and (F) hsa-mir-1234 are shown with (A) nontransfected and (B) empty vector transfected controls.

**Figure S4**


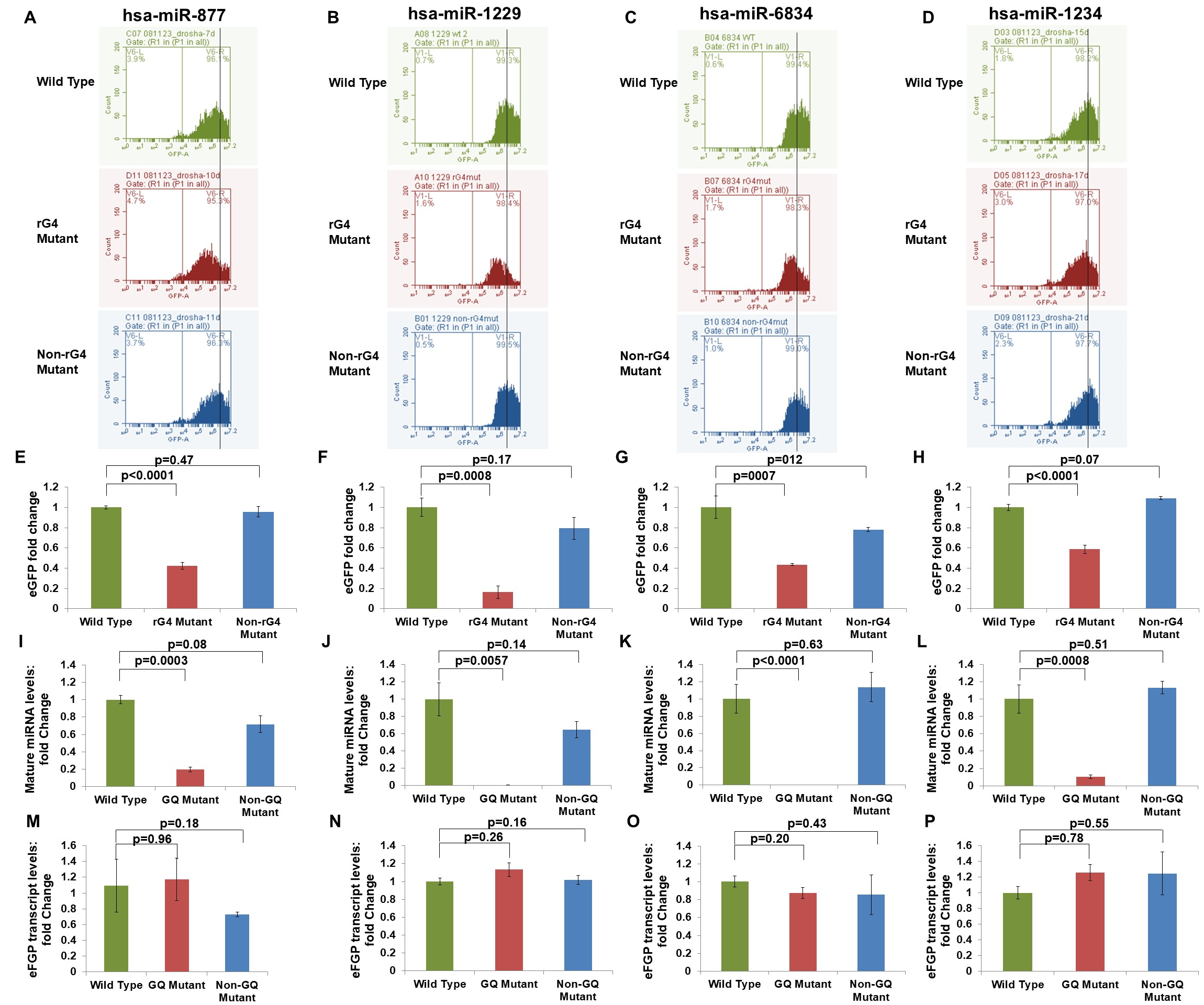


**Figure S4:** Flow cytometry analysis for GFP expression in mirtron wildtype, rG4 mutant and non-rG4 mutant transfected in HCT116 *DROSHA* knockout cell line for (A) hsa-mir-877 (B) hsa-mir-1229 (C) hsa-mir-6834 (D) hsa-mir-1234. Bar graphs showing the median of GFP expression in mirtron wildtype, rG4 mutant and non-rG4 mutant for (E) hsa-mir-877 (F) hsa-mir-1229 (G) hsa-mir-6834 (H) hsa-mir-1234. Differential mature miRNA expression in mirtron wildtype, rG4 mutant and non-rG4 mutant for (I) hsa-mir-877-5’p (J) hsa-mir-1229-5’p (K) hsa-mir-6834-5’p (L) hsa-mir-1234-3’p. Gene expression of eGFP transcripts in mirtron wild type, rG4 mutant and non-rG4 mutant for (M) hsa-mir-877 (N) hsa-mir-1229 (O) hsa-mir-6834 (P) hsa-mir-1234.

**Figure S5:**

**
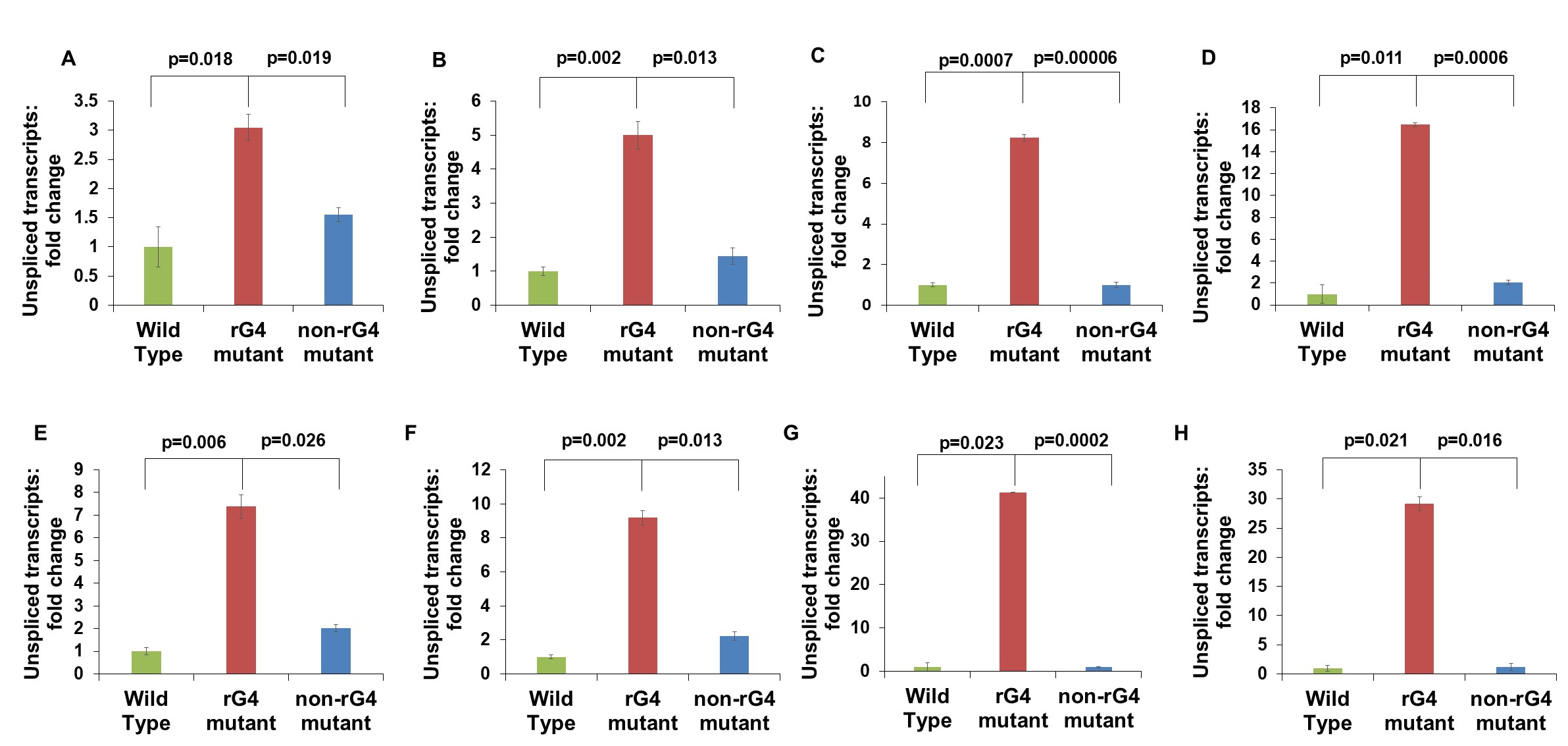
**

**Figure S5: Quantitative assessment of unspliced transcript levels.** Expression of unspliced transcript levels in mirtron wildtype, rG4 mutant and non-rG4 mutant for (A) hsa-mir-877 (B) hsa-mir-1229 (C) hsa-mir-6834 (D) hsa-mir-1234 in HCT116 wild type cell line and in mirtron wild type, rG4 mutant and non-rG4 mutant for (E) hsa-mir-877 (F) hsa-mir-1229 (G) hsa-mir-6834 (H) hsa-mir-1234 in HCT116 *DROSHA* knockout cell line.

**Table S1:** rG4-motif analyses of human mirtrons

| S.No. | Mirtrons | No. of rG4(s) | Position of rG4(s) |
| --- | --- | --- | --- |
| 1 | mir 1224 | 1 | 12 |
| 2 | mir 1226 | 1 | 5 |
| 3 | mir 1227 | 1 | 10 |
| 4 | mir 1228 | 1 | 7 |
| 5 | mir 1229 | 1 | 8 |
| 6 | mir 1233-1 | 2 | 7,28 |
| 7 | mir 1236 | 1 | 13 |
| 8 | mir 1238 | 1 | 23 |
| 9 | mir 6892 | 1 | 11 |
| 10 | mir 877 | 1 | 6 |
| 11 | mir 6765 | 2 | 5, 32 |
| 12 | mir 6789 | 2 | 9, 38 |
| 13 | mir 6800 | 1 | 19 |
| 14 | mir 6807 | 1 | 11 |
| 15 | mir 6821 | 1 | 20 |
| 16 | mir 6831 | 0 | 0 |
| 17 | mir 6834 | 1 | 5 |
| 18 | mir 6844 | 0 | 0 |
| 19 | mir 7108 | 2 | 5, 26 |
| 20 | mir 1237 | 2 | 8, 26 |
| 21 | mir 1292 | 0 | 0 |
| 22 | mir 1976 | 1 | 8 |
| 23 | mir 3605 | 0 | 0 |
| 24 | mir 3606 | 0 | 0 |
| 25 | mir 3620 | 2 | 5, 25 |
| 26 | mir 4649 | 0 | 0 |
| 27 | mir 4667 | 0 | 0 |
| 28 | mir 4701 | 1 | 34 |
| 29 | mir 4723 | 0 | 0 |
| 30 | mir 4728 | 0 | 0 |
| 31 | mir 4742 | 0 | 0 |
| 32 | mir 4743 | 1 | 4 |
| 33 | mir 4750 | 1 | 7 |
| 34 | mir 5004 | 1 | 12 |
| 35 | mir 6515 | 0 | 0 |
| 36 | mir 6728 | 1 | 8 |
| 37 | mir 6733 | 0 | 0 |
| 38 | mir 6738 | 0 | 0 |
| 39 | mir 6739 | 0 | 0 |
| 40 | mir 6745 | 2 | 10, 104 |
| 41 | mir 6747 | 1 | 4 |
| 42 | mir 6758 | 0 | 0 |
| 43 | mir 6784 | 1 | 5 |
| 44 | mir 6786 | 2 | 4, 55 |
| 45 | mir 6788 | 0 | 0 |
| 46 | mir 6798 | 1 | 1 |
| 47 | mir 6802 | 1 | 4 |
| 48 | mir 6806 | 1 | 10 |
| 49 | mir 6810 | 0 | 0 |
| 50 | mir 6820 | 0 | 0 |
| 51 | mir 6837 | 1 | 3 |
| 52 | mir 6843 | 1 | 84 |
| 53 | mir 6856 | 1 | 12 |
| 54 | mir 6860 | 1 | 13 |
| 55 | mir 6863 | 0 | 0 |
| 56 | mir 6889 | 0 | 0 |
| 57 | mir 7106 | 1 | 7 |
| 58 | mir 7107 | 1 | 15 |
| 59 | mir 7109 | 0 | 0 |
| 60 | mir 7114 | 0 | 0 |
| 61 | mir 939 | 1 | 10 |
| 62 | mir 1178 | 1 | 16 |
| 63 | mir 1234 | 1 | 9 |
| 64 | mir 3064 | 0 | 0 |
| 65 | mir 4632 | 1 | 18 |
| 66 | mir 4639 | 0 | 0 |
| 67 | mir 4640 | 1 | 10 |
| 68 | mir 4641 | 1 | 1 |
| 69 | mir 4646 | 1 | 4 |
| 70 | mir 4673 | 1 | 6 |
| 71 | mir 4685 | 1 | 8 |
| 72 | mir 4722 | 1 | 19 |
| 73 | mir 4726 | 1 | 2 |
| 74 | mir 4747 | 1 | 2 |
| 75 | mir 4749 | 1 | 6 |
| 76 | mir 5006 | 1 | 9 |
| 77 | mir 5010 | 0 | 0 |
| 78 | mir 5088 | 1 | 21 |
| 79 | mir 5187 | 0 | 0 |
| 80 | mir 5196 | 1 | 30 |
| 81 | mir 6505 | 0 | 0 |
| 82 | mir 6510 | 0 | 0 |
| 83 | mir 6511a | 0 | 0 |
| 84 | mir 6511b-1 | 1 | 1 |
| 85 | mir 6511b-2 | 0 | 0 |
| 86 | mir 6513 | 0 | 0 |
| 87 | mir 6514 | 0 | 0 |
| 88 | mir 6716 | 0 | 0 |
| 89 | mir 6726 | 1 | 1 |
| 90 | mir 6727 | 1 | 11 |
| 91 | mir 6729 | 1 | 3 |
| 92 | mir 6730 | 0 | 0 |
| 93 | mir 6731 | 1 | 4 |
| 94 | mir 6732 | 1 | 10 |
| 95 | mir 6734 | 0 | 0 |
| 96 | mir 6735 | 1 | 8 |
| 97 | mir 6736 | 1 | 8 |
| 98 | mir 6737 | 1 | 3 |
| 99 | mir 6740 | 0 | 0 |
| 100 | mir 6741 | 1 | 4 |
| 101 | mir 6742 | 0 | 0 |
| 102 | mir 6743 | 2 | 1, 23 |
| 103 | mir 6744 | 1 | 20 |
| 104 | mir 6746 | 1 | 9 |
| 105 | mir 6748 | 1 | 9 |
| 106 | mir 6749 | 1 | 1 |
| 107 | mir 6750 | 0 | 0 |
| 108 | mir 6751 | 1 | 8 |
| 109 | mir 6752 | 1 | 7 |
| 110 | mir 6753 | 0 | 0 |
| 111 | mir 6754 | 1 | 1 |
| 112 | mir 6755 | 0 | 0 |
| 113 | mir 6756 | 1 | 13 |
| 114 | mir 6757 | 1 | 9 |
| 115 | mir 6759 | 0 | 0 |
| 116 | mir 6760 | 0 | 0 |
| 117 | mir 6761 | 0 | 0 |
| 118 | mir 6762 | 0 | 0 |
| 119 | mir 6763 | 0 | 0 |
| 120 | mir 6764 | 0 | 0 |
| 121 | mir 6766 | 0 | 0 |
| 122 | mir 6767 | 0 | 0 |
| 123 | mir 6768 | 1 | 4 |
| 124 | mir 6769a | 1 | 2 |
| 125 | mir 6769b | 1 | 7 |
| 126 | mir 6771 | 1 | 9 |
| 127 | mir 6772 | 1 | 2 |
| 128 | mir 6774 | 0 | 0 |
| 129 | mir 6775 | 1 | 17 |
| 130 | mir 6776 | 1 | 2 |
| 131 | mir 6777 | 1 | 8 |
| 132 | mir 6778 | 1 | 21 |
| 133 | mir 6779 | 1 | 9 |
| 134 | mir 6780a | 0 | 0 |
| 135 | mir 6780b | 1 | 8 |
| 136 | mir 6781 | 1 | 7 |
| 137 | mir 6782 | 1 | 2 |
| 138 | mir 6783 | 0 | 0 |
| 139 | mir 6785 | 1 | 7 |
| 140 | mir 6787 | 1 | 3 |
| 141 | mir 6791 | 1 | 11 |
| 142 | mir 6793 | 0 | 0 |
| 143 | mir 6794 | 1 | 2 |
| 144 | mir 6795 | 1 | 2 |
| 145 | mir 6796 | 1 | 10 |
| 146 | mir 6797 | 0 | 0 |
| 147 | mir 6799 | 1 | 3 |
| 148 | mir 6801 | 1 | 2 |
| 149 | mir 6803 | 1 | 8 |
| 150 | mir 6804 | 1 | 20 |
| 151 | mir 6805 | 1 | 2 |
| 152 | mir 6808 | 1 | 2 |
| 153 | mir 6809 | 0 | 0 |
| 154 | mir 6811 | 0 | 0 |
| 155 | mir 6812 | 1 | 4 |
| 156 | mir 6813 | 1 | 4 |
| 157 | mir 6814 | 0 | 0 |
| 158 | mir 6815 | 1 | 8 |
| 159 | mir 6816 | 1 | 9 |
| 160 | mir 6817 | 1 | 2 |
| 161 | mir 6818 | 0 | 0 |
| 162 | mir 6819 | 1 | 9 |
| 163 | mir 6822 | 0 | 2 |
| 164 | mir 6823 | 1 | 4 |
| 165 | mir 6824 | 1 | 9 |
| 166 | mir 6825 | 1 | 2 |
| 167 | mir 6826 | 1 | 4 |
| 168 | mir 6827 | 0 | 0 |
| 169 | mir 6828 | 0 | 0 |
| 170 | mir 6829 | 0 | 0 |
| 171 | mir 6830 | 1 | 10 |
| 172 | mir 6832 | 0 | 0 |
| 173 | mir 6833 | 1 | 5 |
| 174 | mir 6835 | 0 | 0 |
| 175 | mir 6836 | 1 | 1 |
| 176 | mir 6838 | 0 | 0 |
| 177 | mir 6839 | 0 | 0 |
| 178 | mir 6841 | 0 | 0 |
| 179 | mir 6842 | 0 | 0 |
| 180 | mir 6845 | 0 | 0 |
| 181 | mir 6846 | 1 | 7 |
| 182 | mir 6847 | 1 | 30 |
| 183 | mir 6848 | 1 | 8 |
| 184 | mir 6849 | 1 | 4 |
| 185 | mir 6851 | 1 | 4 |
| 186 | mir 6852 | 0 | 0 |
| 187 | mir 6853 | 1 | 29 |
| 188 | mir 6854 | 0 | 0 |
| 189 | mir 6855 | 0 | 0 |
| 190 | mir 6857 | 0 | 0 |
| 191 | mir 6859 | 1 | 10 |
| 192 | mir 6861 | 1 | 3 |
| 193 | mir 6862 | 0 | 0 |
| 194 | mir 6864 | 0 | 0 |
| 195 | mir 6865 | 1 | 8 |
| 196 | mir 6866 | 0 | 0 |
| 197 | mir 6867 | 0 | 0 |
| 198 | mir 6868 | 0 | 0 |
| 199 | mir 6870 | 1 | 4 |
| 200 | mir 6871 | 1 | 10 |
| 201 | mir 6872 | 1 | 16 |
| 202 | mir 6873 | 0 | 0 |
| 203 | mir 6874 | 0 | 0 |
| 204 | mir 6875 | 1 | 10 |
| 205 | mir 6876 | 1 | 12 |
| 206 | mir 6877 | 0 | 0 |
| 207 | mir 6878 | 0 | 0 |
| 208 | mir 6879 | 1 | 8 |
| 209 | mir 6880 | 1 | 4 |
| 210 | mir 6881 | 1 | 14 |
| 211 | mir 6882 | 0 | 0 |
| 212 | mir 6883 | 1 | 7 |
| 213 | mir 6884 | 0 | 0 |
| 214 | mir 6885 | 0 | 0 |
| 215 | mir 6886 | 0 | 0 |
| 216 | mir 6887 | 1 | 11 |
| 217 | mir 6888 | 0 | 0 |
| 218 | mir 6890 | 1 | 2 |
| 219 | mir 6893 | 1 | 4 |
| 220 | mir 6894 | 1 | 7 |
| 221 | mir 6895 | 0 | 0 |
| 222 | mir 7110 | 1 | 2 |
| 223 | mir 7111 | 1 | 3 |
| 224 | mir 7112 | 0 | 0 |
| 225 | mir 7113 | 0 | 0 |
| 226 | mir 3940 | 1 | 25 |
| 227 | mir 937 | 1 | 13 |
| 228 | mir 1909 | 0 | 0 |
| 229 | mir 4688 | 1 | 44 |
| 230 | mir 4690 | 0 | 0 |
| 231 | mir 4745 | 0 | 0 |
| 232 | mir 4758 | 1 | 15 |
| 233 | mir 6790 | 1 | 19 |
| 234 | mir 6792 | 0 | 0 |
| 235 | mir 6850 | 1 | 17 |
| 236 | mir 6858 | 1 | 5 |
| 237 | mir 6869 | 1 | 10 |
| 238 | mir 6891 | 1 | 5 |
| 239 | mir 1231 | 0 | 0 |

**Table S2:** RNA oligonucleotide sequences of selected human mirtron rG4s

| **S.No.** | **Name** | **Type of rG4** | **RNA Oligonucleotide Sequence** |
| --- | --- | --- | --- |
| 1 | hsa-miR-877 Wildtype | 2G GQ | GGAGAUGGCGCAGGGGACACGG |
| 2 | hsa-miR-877 rG4 Mutant |  | GGAGAUGGCGCAGAAGACACGA |
| 3 | hsa-miR-1229 Wildtype | 3G GQ | GGGUUUGGGGGAGAGCGUGGGCUGGGG |
| 4 | hsa-miR-1229 rG4 Mutant |  | GGGUUUGGAAGAGAGCGUGAGCUGAAG |
| 5 | hsa-miR-6834 Wildtype | 3G GQ | GGGACUGGGAUUUGUGGGGCGAGGAGGG |
| 6 | hsa-miR-6834 rG4 Mutant |  | GGGACUGGGAUUUGUGGAGCGAGAAGAG |
| 7 | hsa-miR-1234 Wildtype | 4G GQ | GGGGUGGCUGGGGCGGGGGGGGCCCGGGG |
| 8 | hsa-miR-1234 rG4 Mutant |  | GGGGUGGCUGAAGCAGAGAGAGCUCGAGA |

**Table S3:** Cloning primer sequences of selected human mirtron rG4s

| Name | Primer Sequence |
| --- | --- |
| hsa-miR-877 Wildtype FP | CAAGGTAGAGGAGATGGCGCAGGGGACACGGGCAAAGACTTGGGGGTTCCTGGGACCCTCAGACGTGTGTCCTCTTCTCCCTCCTCCCAG |
| hsa-miR-877 Wildtype RP | CGTCCTGGGAGGAGGGAGAAGAGGACACACGTCTGAGGGTCCCAGGAACCCCCAAGTCTTTGCCCGTGTCCCCTGCGCCATCTCCTCTAC |
| hsa-miR-877 rG4 Mutant FP | CAAGGTAGAGGAGATGGCGCAGAAGACACGAGCAAAGACTTGGGGGTTCCTGGGACCCTCAGACGTGTGTCCTCTTCTCCCTCCTCCCAG |
| hsa-miR-877 rG4 Mutant RP | CGTCCTGGGAGGAGGGAGAAGAGGACACACGTCTGAGGGTCCCAGGAACCCCCAAGTCTTTGCTCGTGTCTTCTGCGCCATCTCCTCTAC |
| hsa-miR-877 non-rG4 Mutant FP | CAAGGTAGAGGAGATGGCGCAGGGGACACGGGTAAAGACCTGGGGGTTCTTGGGACCCTCAGACGTGTGTCCTCTTCTCCCTCCTCCCAG |
| hsa-miR-877 non-rG4 Mutant RP | CGTCCTGGGAGGAGGGAGAAGAGGACACACGTCTGAGGGTCCCAAGAACCCCCAGGTCTTTACCCGTGTCCCCTGCGCCATCTCCTCTAC |
| hsa-miR-1229 Wildtype FP | CAAGGTGGGTAGGGTTTGGGGGAGAGCGTGGGCTGGGGTTCAGGGACACCCTCTCACCACTGCCCTCCCACAG |
| hsa-miR-1229 Wildtype RP | CGTCCTGTGGGAGGGCAGTGGTGAGAGGGTGTCCCTGAACCCCAGCCCACGCTCTCCCCCAAACCCTACCCAC |
| hsa-miR-1229 rG4 Mutant FP | CAAGGTGGGTAGGGTTTGGAAGAGAGCGTGAGCTGAAGTTCAGAGACACCCTCTCACCACTGCCCTCCCACAG |
| hsa-miR-1229 rG4 Mutant RP | CGTCCTGTGGGAGGGCAGTGGTGAGAGGGTGTCTCTGAACTTCAGCTCACGCTCTCTTCCAAACCCTACCCAC |
| hsa-miR-1229 non-rG4 Mutant FP | CAAGGTGGGCAGGGTCTGGGGGAGAGCGCGGGCTGGGGCTCAGGGACGTCCTCTCACCACTGCCCTCCCACAG |
| hsa-miR-1229 non-rG4 Mutant RP | CGTCCTGTGGGAGGGCAGTGGTGAGAGGACGTCCCTGAGCCCCAGCCCGCGCTCTCCCCCAGACCCTGCCCAC |
| hsa-miR-6834 Wildtype FP | CAAGGTGAGGGACTGGGATTTGTGGGGCGAGGAGGGACCTGTACTAGCCATGGTTCTGATCACATATGTCCCATCCCTCCATCAG |
| hsa-miR-6834 Wildtype RP | CGTCCTGATGGAGGGATGGGACATATGTGATCAGAACCATGGCTAGTACAGGTCCCTCCTCGCCCCACAAATCCCAGTCCCTCAC |
| hsa-miR-6834 rG4 Mutant FP | CAAGGTGAGGGACTGGGATTTGTGGAGCGAGAAGAGACTTGTACTAGCCATAGTTTTGATCACATATGTCCCATCCCTCCATCAG |
| hsa-miR-6834 rG4 Mutant RP | CGTCCTGATGGAGGGATGGGACATATGTGATCAAAACTATGGCTAGTACAAGTCTCTTCTCGCTCCACAAATCCCAGTCCCTCAC |
| hsa-miR-6834 non-rG4 Mutant FP | CAAGGTGAGGGACTGGGATTTGTGGGGTAAGGAGGGTCCTGTACTAGCCATGGCACTGATTACATATGTCCCATCCCTCCATCAG |
| hsa-miR-6834 non-rG4 Mutant RP | CGTCCTGATGGAGGGATGGGACATATGTAATCAGTGCCATGGCTAGTACAGGACCCTCCTTACCCCACAAATCCCAGTCCCTCAC |
| hsa-miR-1234 Wildtype FP | CAAGGTGAGTGTGGGGTGGCTGGGGCGGGGGGGGCCCGGGGACGGCTTGGGCCTGCCTAGTCGGCCTGACCACCCACCCCACAG |
| hsa-miR-1234 Wildtype RP | CGTCCTGTGGGGTGGGTGGTCAGGCCGACTAGGCAGGCCCAAGCCGTCCCCGGGCCCCCCCCGCCCCAGCCACCCCACACTCAC |
| hsa-miR-1234 rG4 Mutant FP | CAAGGTGAGTGTGGGGTGGCTGAAGCAGAGAGAGCTCGAGAACGACTTGAGCTTGCTTAGTCGGCTTGATCACCCACCCCACAG |
| hsa-miR-1234 rG4 Mutant RP | CGTCCTGTGGGGTGGGTGATCAAGCCGACTAAGCAAGCTCAAGTCGTTCTCGAGCTCTCTCTGCTTCAGCCACCCCACACTCAC |
| hsa-miR-1234 non-rG4 Mutant FP | CAAGGTATATGTGGGGTGGTTGGGGCGGGGGGGGCCCGGGGCTGGCTTGGGCCTGCCTGATCGGCCTGACCACCCACCCCACAG |
| hsa-miR-1234 non-rG4 Mutant RP | CGTCCTGTGGGGTGGGTGGTCAGGCCGATCAGGCAGGCCCAAGCCAGCCCCGGGCCCCCCCCGCCCCAACCACCCCACATATAC |
| hsa-miR-6841 Wildtype FP | CAAGGTGTTTAGGGTACTCAGAGCAAGTTGTGAAACACAGGTGTTTTTTAACCTCACCTTGCATCTGCATCCCCAG |
| hsa-miR-6841 Wildtype RP | CGTCCTGGGGATGCAGATGCAAGGTGAGGTTAAAAAACACCTGTGTTTCACAACTTGCTCTGAGTACCCTAAACAC |
| hsa-miR-6888 Wildtype FP | CAAGGTGGGAAGGAGATGCTCAGGCAGATCTGTCTCTGATTGTTTCCAAGATCTGTCTCGATTGTTTCCAG |
| hsa-miR-6888 Wildtype RP | CGTCCTGGAAACAATCGAGACAGATCTTGGAAACAATCAGAGACAGATCTGCCTGAGCATCTCCTTCCCAC |

**Table S4:** qPCR primer sequences

| Name | Primer Sequence |
| --- | --- |
| hsa-miR-877 5’ SL | GTCGTATCCAGTGCAGGGTCCGAGGTATTCGCACTGGATACGACCCCTGC |
| hsa-miR-877 5’ FP | GTAGAGGAGATGGCGCAGG |
| hsa-miR-1229 5’ SL | GTCGTATCCAGTGCAGGGTCCGAGGTATTCGCACTGGATACGACCGCTCT |
| hsa-miR-1229 5’ FP | GTGGGTAGGGTTTGG |
| hsa-miR-6834 5’ SL | GTCGTATCCAGTGCAGGGTCCGAGGTATTCGCACTGGATACGACCCACAA |
| hsa-miR-6834 5’ FP | GTGAGGGACTGGGATTTG |
| hsa-miR-1234 3’ SL | GTCGTATCCAGTGCAGGGTCCGAGGTATTCGCACTGGATACGACGTGGGG |
| hsa-miR-1234 3’ FP | ATTCGGCCTGACCACCCACCC |
| hsa-miR-6841 5’ SL | GTCGTATCCAGTGCAGGGTCCGAGGTATTCGCACTGGATACGACACAACT |
| hsa-miR-6841 5’ FP | TAGGGTACTCAGAGCAAGTT |
| hsa-miR-6888 5’ SL | GTCGTATCCAGTGCAGGGTCCGAGGTATTCGCACTGGATACGACATCTGC |
| hsa-miR-6888 5’ FP | AAGGAGATGCTCAGGCAGA |
| Universal Reverse Primer | CCAGTGCAGGGTCCGAGGTA |
| eGFP-C terminal FP | TATATCATGGCCGACAAGCA |
| eGFP-C terminal RP | GAACTCCAGCAGGACCATGT |
| Unspliced transcript FP | CTACGGCAAGCTGACCCT |
| hsa-mir-877 pre-miRNA RP |  |
| hsa-mir-1229 pre-miRNA RP | CTGTGGGAGGGCAGTGGTGA |
| hsa-mir-6834 pre-miRNA RP | CTGATGGAGGGATGGGACATA |
| hsa-mir-1234 pre-miRNA RP | CTGTGGGGTGGGTGGTCA |
| GAPDH FP | TGCACCACCAACTGCTTAGC |
| GAPDH RP | GGCATGGACTGTGGTCATGAG |
